# Genome-wide association meta-analysis of fish and EPA+DHA consumption in 17 US and European cohorts

**DOI:** 10.1371/journal.pone.0186456

**Published:** 2017-12-13

**Authors:** Dariush Mozaffarian, Hassan S Dashti, Mary K Wojczynski, Audrey Y Chu, Jennifer A Nettleton, Satu Männistö, Kati Kristiansson, Mägi Reedik, Jari Lahti, Denise K Houston, Marilyn C Cornelis, Frank J. A van Rooij, Maria Dimitriou, Stavroula Kanoni, Vera Mikkilä, Lyn M Steffen, Marcia C de Oliveira Otto, Lu Qi, Bruce Psaty, Luc Djousse, Jerome I Rotter, Kennet Harald, Markus Perola, Harri Rissanen, Antti Jula, Fischer Krista, Evelin Mihailov, Mary F Feitosa, Julius S Ngwa, Luting Xue, Paul F Jacques, Mia-Maria Perälä, Aarno Palotie, Yongmei Liu, Nike A Nalls, Luigi Ferrucci, Dena Hernandez, Ani Manichaikul, Michael Y Tsai, Jessica C Kiefte-de Jong, Albert Hofman, André G Uitterlinden, Loukianos Rallidis, Paul M Ridker, Lynda M Rose, Julie E Buring, Terho Lehtimäki, Mika Kähönen, Jorma Viikari, Rozenn Lemaitre, Veikko Salomaa, Paul Knekt, Andres Metspalu, Ingrid B Borecki, L. Adrienne Cupples, Johan G Eriksson, Stephen B Kritchevsky, Stefania Bandinelli, David Siscovick, Oscar H Franco, Panos Deloukas, George Dedoussis, Daniel I Chasman, Olli Raitakari, Toshiko Tanaka

**Affiliations:** 1 Friedman School of Nutrition Science & Policy, Tufts University, Boston, MA, United States of America; 2 Nutrition and Genomics Laboratory, Jean Mayer US Department of Agriculture Human Nutrition Research Center on Aging, Tufts University, Boston, MA, United States of America; 3 Division of Statistical Genomics, Department of Genetics, Washington University School of Medicine, St. Louis, MO, United States of America; 4 Division of Preventive Medicine, Brigham and Women's Hospital, Boston, MA, United States of America; 5 Division of Epidemiology, Human Genetics and Environmental Sciences, University of Texas Health Science Center at Houston, Houston, TX, United States of America; 6 National Institute for Health and Welfare, Helsinki, Finland; 7 Estonian Genome Center, University of Tartu, Tartu, Estonia; 8 Institute of Behavioural Sciences, University of Helsinki, Helsinki, Finland; 9 Folkhälsan Research Centre, Helsinki, Finland; 10 Sticht Center on Aging, Wake Forest School of Medicine, Winston Salem, NC, United States of America; 11 Department of Nutrition, Harvard School of Public Health, Boston, MA, United States of America; 12 Department of Epidemiology, Erasmus MC, Rotterdam, The Netherlands; 13 Netherlands Genomics Initiative-sponsored Netherlands Consortium for Healthy Aging, Leiden, The Netherlands; 14 Department of Dietetics and Nutrition, Harokopio University, Athens, Greece; 15 William Harvey Research Institute, Barts and The London School of Medicine and Dentistry, Queen Mary University of London, London, United Kingdom; 16 Department of Food and Environmental Sciences, University of Helsinki, Finland; 17 Research Centre of Applied and Preventive Cardiovascular Medicine, University of Turku, Turku, Finland; 18 Division of Epidemiology and Community Health, University of Minnesota School of Public Health, Minneapolis, MN, United States of America; 19 Department of Medicine, Epidemiology and Health Services, University of Washington, Seattle, WA, United States of America; 20 Department of Medicine, Harvard Medical School, and Division of Aging Brigham and Women's Hospital, Boston, MA, United States of America; 21 Los Angeles Biomedical Research Institute and Department of Pediatrics, Harbor-UCLA Medical Center, Los Angeles, CA, United States of America; 22 Institute for Molecular Medicine Finland (FIMM), University of Helsinki, Helsinki, Finland; 23 University of Tartu, Estonian Genome Center, Tartu, Estonia; 24 Division of Statistical Genomics, Department of Genetics, Washington University School of Medicine, St. Louis, MO, United States of America; 25 Department of Biostatistics, Boston University School of Public Health, Boston, MA, United States of America; 26 Division of Cardiovascular Medicine, Howard University College of Medicine, Washington DC, United States of America; 27 Nutritional Epidemiology Program, USDA Human Nutrition Research Center on Aging, Tufts University, Boston, MA, United States of America; 28 Department of Chronic Disease Prevention, National Institute for Health and Welfare, Helsinki, Finland; 29 Department of Medical Genetics, University of Helsinki and University Central Hospital, Helsinki, Finland; 30 Department of Biostatistical Sciences, Wake Forest School of Medicine, Winston Salem, NC, United States of America; 31 Laboratory of Neurogenetics, National Institute of Aging, Bethesda, MD, United States of America; 32 Clinical Research Branch, National Institute on Aging, Baltimore, MD, United States of America; 33 Center for Public Health Genomics and Division of Biostatistics and Epidemiology, Department of Public Health Sciences, University of Virginia, Charlottesville, VA, United States of America; 34 Department of Laboratory Medicine and Pathology, University of Minnesota, Minneapolis, MN, United States of America; 35 Department of Epidemiology, Erasmus MC, Rotterdam, The Netherlands; 36 Leiden University College, The Hague, The Netherlands; 37 Department of Internal Medicine, Erasmus MC, Rotterdam, The Netherlands; 38 Second Department of Cardiology, University General Hospital Attikon, Athens, Greece; 39 Harvard Medical School, Boston MA, United States of America; 40 Department of Clinical Chemistry, Fimlab Laboratories, University of Tampere School of Medicine, Tampere, Finland; 41 Department of Clinical Physiology, University of Tampere and Tampere University Hospital, Tampere, Finland; 42 Department of Medicine, University of Turku and Turku University Hospital, Turku, Finland; 43 Department of Medicine, University of Washington, Seattle, WA, United States of America; 44 NHLBI Framingham Heart Study, Framingham, MA, United States of America; 45 Department of General Practice and Primary health Care, University of Helsinki, Helsinki, Finland; 46 Helsinki University Central Hospital, Unit of General Practice, Helsinki, Finland; 47 Geriatric Rehabilitation Unit, Azienda Sanitaria Firenze, Florence, Italy; 48 New York Academy of Medicine, New York, NY, United States of America; 49 Princess Al-Jawhara Al-Brahim Centre of Excellence in Research of Hereditary Disorder, King Abdulaziz University, Jeddah, Saudi Arabia; 50 Department of Clinical Physiology and Nuclear Medicine, Turku University Hospital, Turku, Finland; Vrije Universiteit Amsterdam, NETHERLANDS

## Abstract

**Background:**

Regular fish and omega-3 consumption may have several health benefits and are recommended by major dietary guidelines. Yet, their intakes remain remarkably variable both within and across populations, which could partly owe to genetic influences.

**Objective:**

To identify common genetic variants that influence fish and dietary eicosapentaenoic acid plus docosahexaenoic acid (EPA+DHA) consumption.

**Design:**

We conducted genome-wide association (GWA) meta-analysis of fish (*n* = 86,467) and EPA+DHA (*n* = 62,265) consumption in 17 cohorts of European descent from the CHARGE (Cohorts for Heart and Aging Research in Genomic Epidemiology) Consortium Nutrition Working Group. Results from cohort-specific GWA analyses (additive model) for fish and EPA+DHA consumption were adjusted for age, sex, energy intake, and population stratification, and meta-analyzed separately using fixed-effect meta-analysis with inverse variance weights (METAL software). Additionally, heritability was estimated in 2 cohorts.

**Results:**

Heritability estimates for fish and EPA+DHA consumption ranged from 0.13–0.24 and 0.12–0.22, respectively. A significant GWA for fish intake was observed for rs9502823 on chromosome 6: each copy of the minor allele (Freq_A_ = 0.015) was associated with 0.029 servings/day (~1 serving/month) lower fish consumption (P = 1.96x10^-8^). No significant association was observed for EPA+DHA, although rs7206790 in the obesity-associated FTO gene was among top hits (*P* = 8.18x10^-7^). Post-hoc calculations demonstrated 95% statistical power to detect a genetic variant associated with effect size of 0.05% for fish and 0.08% for EPA+DHA.

**Conclusions:**

These novel findings suggest that non-genetic personal and environmental factors are principal determinants of the remarkable variation in fish consumption, representing modifiable targets for increasing intakes among all individuals. Genes underlying the signal at rs72838923 and mechanisms for the association warrant further investigation.

## Introduction

Consumption of fish (including finfish and shellfish) and long-chain omega-3 fatty acids is linked to lower risk of several chronic diseases, in particular fatal coronary heart disease [1]. These beneficial associations in observational studies are supported by randomized controlled trials demonstrating favorable effects of fish or fish oil on numerous chronic disease risk factors and on cardiac mortality [1,2]. As a result, regular fish consumption is recommended by all major national and international dietary guidelines [1].

In contrast to these guidelines and in comparison to many other foods, remarkable variation exists in the amount of fish consumption within and across populations. In many Western nations, approximately one-third of individuals consume no fish at all, approximately one-third consume fish but relatively rarely (up to once per week), and approximately one-third consume fish more frequently [3]. While some of this wide variation in fish consumption is undoubtedly due to personal and environmental factors (e.g., culture, geographic residence, family habits, socioeconomic status), the potential contribution of intrinsic biologic factors, such as genetic variation, is not well established. In one analysis among Danish twins, the estimated heritability of fish consumption was 17% in men and 61% in women, based on additive genetic effects [4]. For example, potential heritability could relate to differences in genes related to taste, digestion, fatty acid metabolism, or other unknown processes related to food preferences. Yet, the potential genetic variants underlying this estimated heritability are unknown; and such heritability estimates also require further replication.

A basic concept underlying “personalized nutrition” is that a person’s genes can influence their behaviors and responses to the environment. Dietary habits, including the consumption of fish, are among the most relevant factors that influence the development of chronic diseases. Elucidating whether, and in what manner, specific genes alter fish and long-chain omega-3 fatty acid consumption would have implications for understanding influences on variation in fish intake within populations and the biology of partiality to foods. Furthermore, identification of such variants could also inform the development of personalized nutrition—dietary recommendations based on genetic preferences for consumption.

As has been seen with other characteristics such as physiologic risk factors, genome-wide association (GWA) studies may lead to discovery of novel genes and biologic pathways that influence the individual characteristic of interest. Although such studies have been performed for major macronutrients (e.g., fat, carbohydrate, protein) [5,6], few analyses have been done for specific foods[7], whose intakes may be influenced by complex characteristics of tastes, textures, aromas, and nutrient contents. The ability to undertake food-specific genetic analyses has been limited by the modest sample sizes of individual cohorts having both dietary and genetic information and the potential lack of reproducibility of genetic findings discovered in any single cohort.

We therefore performed a collaborative investigation to estimate heritability of and assess how common genetic variation relates to dietary consumption of both fish and long-chain omega-3 fatty acids (eicosapentaenoic acid (EPA) and docosahexaenoic acid (DHA)) as part of the of the CHARGE (Cohorts for Heart and Aging Research in Genomic Epidemiology) Consortium Nutrition Working Group, bringing together investigators and data from 17 US and European population-based cohort studies totaling 86,467 participants of European descent.

## Subjects and methods

### Cohorts

The present work was a collaboration among 17 US and European population-based cohort studies participating in the Nutrition Working Group of the CHARGE Consortium (**S1 Table**). These included the Atherosclerosis Risk in Communities Study (ARIC); Cardiovascular Health Study (CHS); Dietary, Lifestyle, and Genetic Determinants of Obesity and Metabolic Syndrome (DILGOM); Estonian Study; Family Heart Study (FamHS); Framingham Heart Study (FHS); Helsinki Birth Cohort Study (HBCS); Health 2000 survey (H2000); Health, Aging, and Body Composition (HealthABC) Study; Health Professionals Follow-up Study (HPFS); Invecchiare [Aging] in Chianti Area (InCHIANTI); Multi-Ethnic Study of Atherosclerosis (MESA); Nurses’ Health Study (NHS); Rotterdam Study; The Hellenic Study of Interactions between SNPs and Eating in Atherosclerosis Susceptibility (THESIAS); Women’s Genome and Health study (WGHS); and Young Finns Study (YFS). Additional details on these cohorts have been published previously [5,6] and are provided in S1 Table. All persons studied were of European descent, consented to genetic research, and provided written informed consent. For each study, examination protocols were approved by local institutional review boards at Johns Hopkins University (ARIC, MESA), University of Washington (CHS), Epidemiology and Public Health of the Hospital District of Helsinki and Uusimaa (DILGOM), Washington University (FamHS), Boston University (FHS), University of Pittsburgh (HealthABC), Harvard University (HPFS, NHS), National Public Health Institute of Finland (HBCS), Epidemiology and Public Health of the Hospital District of Helsinki and Uusimaa (H2000), Italian National Institute of Research and Care of Aging (InCHIANTI), Erasmus Medical Center and The Netherlands Ministry of Health, Welfare and Sports (Rotterdam), Harokopio University (THESIAS), Brigham and Women’s Hospital (WGHS), University of Helsinki (YFS), and procedures were in accordance with the ethical standards of the responsible institutional or regional committee on human subject research.

### Assessment of fish and omega-3 fatty acid consumption

Usual dietary intake was assessed in each cohort using detailed food frequency questionnaires designed to capture the dietary habits of the population under study (**S2 Table**). Typically, participants were asked to indicate how often, on average, they had consumed various foods and beverages over the past year according to multiple frequency categories (e.g., 9 categories ranging from <1/month to 6+/day), with usual portion sizes specified on the questionnaire or by the participant. Fish intake was generally assessed using multiple questions, such as on consumption of tuna fish; dark meat fish such as salmon or sardines; other white fish; shellfish; and fried fish or fish sandwiches. For each question, the midpoint of each frequency category was used to estimate usual intake which was then multiplied by the specified portion size; these intakes were summed across all questions on fish. For this analysis, we standardized fish consumption in each cohort to 100g servings/day. In 12 cohorts, total dietary consumption of eicosapentaenoic acid (EPA) and docosahexaenoic acid (DHA) was estimated by linking the dietary assessment tool to a food composition table specific to the cohort (e.g., the USDA food composition database in the US). For each of the types of foods consumed, the frequency and average portion size were multipled by the content of EPA/DHA in the food. The total was calculated by summing across all foods in the questionnaire. For cohorts that included nutrients from supplements, the portion of EPA+DHA from supplements was excluded from our analysis.

### Heritability estimates

To evaluate potential heritability of fish and EPA+DHA consumption, we estimated heritability using family-based methods in two family-based cohorts (FamHS and FHS) using the variance components method in Sequential Oligogenic Linkage Analysis Routines (SOLAR; Texas Biomedical Research Institute; San Antonio, TX), and adjusting for age and sex. Briefly, heritability is calculated using a maximum likelihood method using the ratio of the genetic variance to total phenotypic variance.[8]

### Genotyping and analysis

Genome-wide genotyping was conducted in each cohort using Affymetrix or Illumina platforms. Each study performed quality control for genotyped single nucleotide polymorphisms (SNPs) based on minor allele frequency (MAF), call rate, and departure from Hardy-Weinberg equilibrium (**S3 Table**). Phased haplotypes from HapMap CEU were used to impute ~2.5 million autosomal SNPs using a Hidden Markov Model algorithm implemented in MACH, IMPUTE, or BimBam. Study-specific GWA analyses were conducted within each cohort using genotyped and imputed SNP dosages assuming an additive genetic model. Fish and EPA+DHA consumption were separately evaluated as the dependent variable using linear regression with robust standard error, adjusted for age, sex, energy intake (kcal/d), study-specific centers where applicable, and population stratification principal components when the cohort lambda was >1. SNPs with low MAF (<1%), low imputation quality (MACH: R^2^<0.3; or IMPUTE: proper info <0.4), were excluded. Quality control for cohort-level GWAS results was performed to ensure correct specification of the minor allele and agreement in frequencies with the reference population (HapMap CEU), consistent distribution of effect sizes and standard error, and examination of QQ plots to assess any large inflation of test statistics. Results across studies were combined using fixed-effect meta-analysis with inverse variance weights (METAL software)[9]. The association results from individual studies as well as meta-analyses were adjusted for genomic control. To explore potential heterogeneity by demographic region, a meta-analysis within cohorts from Europe and USA was performed. Genome-wide significance was considered at the Bonferroni-corrected threshold of *P*<5x10^-8^. Statistical power to detect a true association at various effect sizes (heritability) was calculated using GWAPower software for the analysis of 1 million independent SNPs for both fish and EPA+DHA (Feng S, 2011 BMC Genetics), assuming linkage disequilibrium (*r*^2^) of 0.5 between a SNP and putative causal variant and 10% variance explained by three covariates.

### Exploratory analysis of plasma phospholipid EPA and DHA

Result from genome-wide association analyses of circulating EPA and DHA are publically available (http://faculty.washington.edu/rozenl/files/) [10]. These databases were mined to test whether the top SNPs from the fish and EPA+DHA intake GWAS are associated with circulating levels of EPA and DHA.

## Results

The 17 cohorts were from the US, Estonia, Finland, Greece, Italy, and the Netherlands and included 86,467 participants with information on fish consumption and 62,265 with information on EPA+DHA consumption. Across participating cohorts, mean fish consumption ranged from 0.19 servings/day (FHS) to 0.75 servings/day (THISEAS) (**Table 1**). Mean intake of EPA+DHA consumption ranged from 89 (Rotterdam) to 563 (HBCS) mg/d and was generally consistent with findings on fish intake, except in THISEAS (Greece) which had relatively higher intakes of fish than EPA+DHA, suggesting predominant consumption of white (non-oily) fish. In general, participants in European cohorts had higher fish consumption than those in US cohorts.

**Table 1:** Characteristics of the cohorts included in this analysis of the genetics of fish consumption.

Study	Maximum N	Age	% Female	Total Fish Intake (serv/day) Median (5-95th%)		Dietary EPA+DHA (mg/d) Median (5-95th%)	
**ARIC**	9557	54.3 ± 5.7	53	0.21	(0.0–0.9)	180	(10–730)
**CHS**	3190	72.3 ± 5.4	61	0.29	(0.1–0.8)	191	(27–569)
**DILGOM_METABO**	3467	51.5 ± 13.4	55	0.42	(0.1–1.3)	431	(119–1277)
**DILGOM_GWA**	604	52.4 ± 13.5	52	0.44	(0.1–1.2)	444	(107–1233)
**ESTONIAN Study**	9920	48.8 ± 20.1	53	0.21	(0.0–0.6)	-	-
**FamHS**	3640	52.2 ± 13.7	53	0.14	(0.0–0.7)	170	(1–680)
**FHS**	7044	47.3 ± 11.8	54	0.13	(0.0–0.6)	200	(40–640)
**Health ABC**	1494	74.8 ± 2.9	48	0.20	(0.2–0.3)	-	-
**Health 2000**	1935	50.5 ± 10.9	51	0.39	(0.1–1.1)	505	(102–1477)
**HBCS**	1701	61.5 ± 2.9	57	0.44	(0.1–1.3)	563	(145–1895)
**HPFS**	4133	58.6 ± 8.7	0	0.29	(0.1–0.9)	-	-
**InCHIANTI**	1194	68.3 ± 15.4	55	0.19	(0.0–0.5)	-	-
**MESA**	2305	62.7 ± 10.2	52	0.17	(0.0–0.7)	100	(20–300)
**NHS**	6776	54.4 ± 6.7	100	0.28	(0.1–0.8)	-	-
**Rotterdam**	4606	67.6 ± 7.7	59	0.07	(0.0–0.5)	89	(8–443)
**THISEAS**	395	59.4 ± 13.0	41	*0*.*59*	(0.0–2.0)	137	(4–479)
**WGHS**	22691	54.7 ± 7.1	100	*0*.*20*	(0.0–0.7)	150	(30–470)
**YFS**	1815	37.8 ± 5.0	56	0.34	(0.1–0.9)	357	(92–902)

Abbreviations: Atherosclerosis Risk in Communities Study (ARIC); Cardiovascular Health Study (CHS); Dietary, Lifestyle, and Genetic Determinants of Obesity and Metabolic Syndrome (DILGOM); Estonian Study; Family Heart Study (FamHS); Framingham Heart Study (FHS); Helsinki Birth Cohort Study (HBCS); Health 2000 survey (H2000); Health, Aging, and Body Composition (HealthABC) Study; Health Professionals Follow-up Study (HPFS); Invecchiare [Aging] in Chianti Area (InCHIANTI); Multi-Ethnic Study of Atherosclerosis (MESA); Nurses’ Health Study (NHS); Rotterdam Study; The Hellenic Study of Interactions between SNPs and Eating in Atherosclerosis Susceptibility (THESIAS); Women’s Genome and Health study (WGHS); and Young Finns Study (YFS).

**Fig 1:**
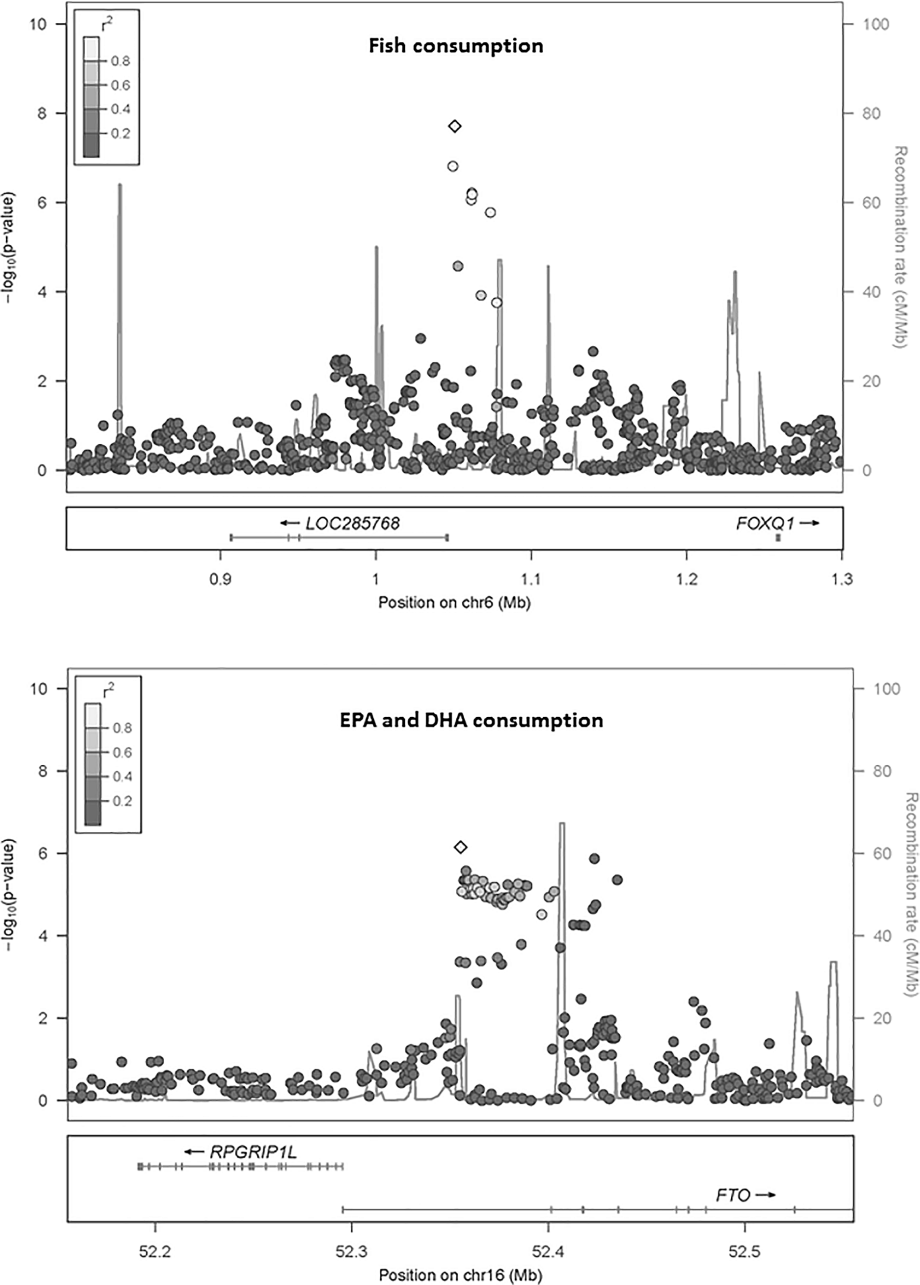
Regional association plot. The figures display–log10 p-values for SNPs within the locus of highest significance for the analysis of fish consumption (top panel) and eicosapentaenoic acid (EPA) and docosahexaenoic acid (DHA) consumption (bottom panel). The degree of linkage disequilibrium (r^2^) is displayed in gradients of gray from dark (low linkage) to light (high linkage).

The heritability estimates for fish intake were 0.13±0.03 (FamHS) and 0.24±0.02 (FHS); and for EPA+DHA intake, 0.12±0.03 (FamHS) and 0.22±0.02 (FHS). In GWA meta-analyses of fish (17 cohorts) and EPA+DHA (11 cohorts) consumption, the genomic control lambda values were 1.07 and 0.99 respectively (**S1 and S2 Figs**). A genome-wide significant association was observed for fish intake on chromosome 6 for rs9502823 (**Table 2**). The minor allele (Freq_A_ = 0.015) was associated with 0.029 servings/day lower fish consumption (P = 1.96x10^-8^). This SNP was mapped to LOC285768 gene of unknown function (**Fig 1,** top panel); and was not identified in NHGRI-EBI GWAS catalogue (http://www.ebi.ac.uk/gwas/, search on Feb 23, 2017). The second top hit was rs17396472 on chromosome 3, not achieving genome-wide statistical significance (P = 5.62x10^-8^).

**Table 2:** The most significant associations from genome-wide association meta-analysis of fish and EPA+DHA consumption.

Trait	SNPID	Chr	Position	N	Effect/ Non-effect	Freq (Effect)	Effect	StdErr	P-value
Fish Intake	rs9502823	6	1050674	71910	A/G	0.02	-0.029	0.005	1.96E-08
(serving/day)	rs17396472	3	68469758	63102	A/T	0.98	0.031	0.005	5.62E-08
	rs1860343	12	4583970	78153	T/C	0.52	-0.006	0.001	4.30E-07
	rs1562806	15	35038800	61909	T/C	0.94	0.022	0.004	1.41E-06
	rs16834168	1	150754770	77898	A/G	0.02	-0.020	0.004	1.58E-06
EPA+DHA	rs11877506	18	49176846	52909	A/G	0.96	16.313	3.091	1.18E-07
(mg/day)	rs2456163	19	1427432	36937	T/C	0.04	-15.560	3.087	4.20E-07
	rs7476409	10	24809042	52909	T/C	0.05	-17.907	3.589	5.50E-07
	rs7206790	16	52355409	56099	C/G	0.55	-7.000	1.420	7.44E-07

No genome-wide significant association was observed for EPA+DHA consumption (**S1 and S2 Figs**). The top association for EPA+DHA consumption was observed for rs11877506 (*P* = 1.18x10^-7^) (Table 2). Additionally, rs7206790 in the obesity-associated FTO gene was among the top SNPs for EPA+DHA intake: the body mass index-raising G allele was associated with 7mg/day greater EPA+DHA intake (**Fig 1,** bottom panel; P = 7.44x10^-7^).

To obtain more information on the locus associated with fish consumption on chromosome 6, we investigated data from the ENCODE project. Using CEU 1000genomes data, we calculated the LD within the region 250kb upstream and downstream from rs9502823. Mapping the SNPs found in the rs9502823 LD block to ENCODE regulatory regions, we identified rs72838923 (in complete LD with rs9502823) as a functional candidate. rs72838923 falls within a experimentally determined H3k27Ac region, identified in several cell types in ENCODE. H3k27Ac regions are thought to be markers of active enhancer activity. In addition, rs72838923 falls within DNAse Hypersensitivity Peak which were identified experimentally across 65 cell types from the ENCODE project. There is further evidence of transcription factor binding sites for FOXA1, among others in this region, from ENCODE CHIP-Seq experiments. In addition, mapping rs72838923 on the UCSC genome browser suggested that this SNP is found within a region of conservation across mammals.

Due to the varying ranges of average fish consumption in US versus European studies, we performed exploratory subgroup GWA meta-analysis stratified by geographic location. No significant associations were identified in USA nor European studies (**S3 Fig**) for fish or EPA+DHA consumption (**S4 Fig**).

A prior consortium analysis including several of these same cohorts reported on genome-wide association of SNP variants with plasma phospholipid EPA and DHA, the concentrations of which are determined by both dietary intake and endogenous metabolism regulation.[11] In exploratory analysis, we evaluated whether the top 5 hits for fish consumption and the top 4 hits for estimated dietary EPA+DHA consumption identified in the present analysis were associated with plasma phospholipid concentrations of EPA or DHA in that prior analysis [10], adjusting for multiple comparisons (9 SNPs x 2 fatty acids = Bonferroni-corrected alpha of 0.05/18 = 0.0028). No significant associations were identified (**S4 Table**).

## Discussion

In this large GWA meta-analysis of 17 US and European cohorts totaling 86,467 participants, we found evidence that common genetic variation may be associated with consumption of fish. We found no genome-wide significant association of common variants with EPA+DHA intake. While the sample size for the analysis of EPA+DHA was smaller than the fish intake analysis, with 62,265 individuals the analysis had 95% power to detect an effect size (heritability) of 0.08%.

We identified one locus on chromosome 6 in association with fish consumption. The SNP was mapped to *LOC285768* with unknown function. The next closest gene is forkhead box Q1 (*FOXQ1*) which is a member of the cancer-associated forkhead-box (FOX) gene family [12]. Our evaluation of data from ENCODE, taken together, identified a functional candidate, rs72838923, that appears to lie within a transcriptionally active region of the genome. While the association was statistically significant, the magnitude of effect was small, with the minor allele being associated with a difference of 0.03 servings/day or approximately 1 serving/month of fish. This finding is more likely to be relevant for understanding the biology of food preferences than for influencing clinical outcomes, although even small differences in fish consumption, over a lifetime, could influence health. The nonsignificant top associations identified in chromosomes 1, 3, and 12 each represent intragenic regions of genes highly expressed in the brain, but these associations did not achieve genome-wide significance.

In heritability analyses, we found evidence for modest heritability of fish (0.13 to 0.24) and EPA+DHA (0.12 to 0.22). Our GWA results identified one locus in association with fish intake that cannot fully account for this observed heritability, suggesting that observed heritability might be due to remarkably small effects across a large number of SNPs, other types of genetic variation such as copy number variants, epigenetic modifications, or multiple unobserved genetic interactions with unknown environmental factors. This challenge of “missing” or unaccounted for heritability is a frequent finding in GWA analyses of common diseases and traits [13]. Heritability analyses may overestimate heritability due to unmeasured shared environmental influences, for example from *in utero*/placental influences through childhood and adult life. In this light, our heritability findings are lower than those previously reported [4] and represent an additional important new contribution. Our findings support the need for future investigations of the possible explanations for the modest but as yet missing heritability of fish and EPA+DHA consumption.

This investigation had several strengths. Our pooling of multiple large, well-established cohorts provided a very large sample of participants for investigating our research questions. Our post-hoc power calculations demonstrate 95% statistical power to detect a genetic variant associated with an effect size of 0.05% for fish consumption and 0.08% for EPA+DHA consumption. We adjusted for total reported energy intake, which helps to address any systematic over- or under-reporting by individuals and also real differences in total food consumed (i.e., due to differences in age, sex, body size, or physical activity), facilitating evaluation of dietary composition. All the studies in the meta-analysis used comparable dietary assessment tools that were appropriate for the population under study, providing the highest quality data that can be reasonably collected across multiple large epidemiological studies.

Limitations should be considered. While dietary intakes assessed by food frequency questionnaire represent a reasonably valid method to collect data on usual dietary habits in large populations [14], such data also include measurement error, which could limit the ability to detect true associations. However, many validation studies have demonstrated that fish and EPA+DHA consumption are measured reasonably well by food frequency questionnaires, whether compared with multiple diet records or with objective circulating or tissue biomarkers [15,16,17,18]. Indeed, because biomarker levels also represent imperfect measures of “true” habitual consumption with uncorrelated errors compared to questionnaire estimates, the actual correlations of estimated fish or EPA+DHA consumption with “true” consumption are likely much higher, in the range of 0.8 or more. Compared with a candidate gene approach, GWA has lower statistical power to detect small genetic effects. Yet, candidate gene approaches for evaluating fish consumption would be strongly limited by imperfect knowledge of which genes affect known systems and biologic processes related to food preferences and, even more so, which genes may affect currently unknown systems and biologic influences on food preferences.

In summary, this large pooling project across 17 established cohorts identified modest heritability of fish and omega-3 fatty acid consumption and one genetic locus associated with fish consumption. These findings suggest that genetic variation may have small effects on fish consumption and, by extension, that other modifiable factors–for example, childhood diet, culture, education, income, and local availability–are the main determinants of the remarkable differences in fish consumption within and across populations, representing targets for increasing fish intake among all individuals.

## Supporting information

S1 Fig Quantile-quantile plots for (A) fish consumption and (B) EPA+DHA consumption. (TIFF)

S2 Fig Genome-wide scans. Genome-wide association meta-analysis results of fish (A) and eicosapentanoic acid and docasahexanoic acid (B) with ~2.5 million SNPs graphed by chromosome position and–log10 p-value.
(TIFF)

S3 Fig Genome-wide scans of fish intake by geographic location. Genome-wide association meta-analysis results of fish consumption in European cohorts (A) and cohorts from the United States (B) for ~2.5 million SNPs graphed by chromosome position and–log10 p-value.
(TIFF)

S4 Fig Genome-wide scans of EPA+DHA intake by geographic location. Genome-wide association meta-analysis results of EPA+DHA consumption in European cohorts (A) and cohorts from the United States (B) for ~2.5 million SNPs graphed by chromosome position and–log10 p-value.
(TIFF)

S1 Table Description of cohorts from the CHARGE consortium. (DOCX)

S2 Table Dietary assessment methods for CHARGE cohorts. (DOCX)

S3 Table Genotyping methods for CHARGE cohorts. (DOCX)

S4 Table Associations of top Fish and EPA+DHA SNPs with circulating DHA and EPA levels. (DOCX)

S1 File Cohort Acknowledgments. (DOCX)

## Abbreviations

EPA: eicosapentaenoic acid
DHA: docosahexaenoic acid
LD: linkage disequilibrium
GWAS: genome-wide association study

## References

[1] Mozaffarian D, Wu JH (2011) Omega-3 fatty acids and cardiovascular disease: effects on risk factors, molecular pathways, and clinical events. J Am Coll Cardiol 58: 2047–2067. doi: 10.1016/j.jacc.2011.06.063 22051327 10.1016/j.jacc.2011.06.063

[2] Rizos EC, Ntzani EE, Bika E, Kostapanos MS, Elisaf MS (2012) Association between omega-3 fatty acid supplementation and risk of major cardiovascular disease events: a systematic review and meta-analysis. JAMA 308: 1024–1033. doi: 10.1001/2012.jama.11374 22968891 10.1001/2012.jama.11374

[3] Papanikolaou Y, Brooks J, Reider C, Fulgoni VL, 3rd (2014) U.S. adults are not meeting recommended levels for fish and omega-3 fatty acid intake: results of an analysis using observational data from NHANES 2003–2008. Nutr J 13: 31 doi: 10.1186/1475-2891-13-31 24694001 10.1186/1475-2891-13-31 PMC3992162

[4] Hasselbalch AL, Heitmann BL, Kyvik KO, Sorensen TI (2008) Studies of twins indicate that genetics influence dietary intake. J Nutr 138: 2406–2412. doi: 10.3945/jn.108.087668 19022965 10.3945/jn.108.087668

[5] Tanaka T, Ngwa JS, van Rooij FJ, Zillikens MC, Wojczynski MK, Frazier-Wood AC, et al (2013) Genome-wide meta-analysis of observational studies shows common genetic variants associated with macronutrient intake. Am J Clin Nutr 97: 1395–1402. doi: 10.3945/ajcn.112.052183 23636237 10.3945/ajcn.112.052183 PMC3652928

[6] Chu AY, Workalemahu T, Paynter NP, Rose LM, Giulianini F, Tanaka T, et al (2013) Novel locus including FGF21 is associated with dietary macronutrient intake. Hum Mol Genet 22: 1895–1902. doi: 10.1093/hmg/ddt032 23372041 10.1093/hmg/ddt032 PMC3612009

[7] Amin N, Byrne E, Johnson J, Chenevix-Trench G, Walter S, Nolte IM, et al (2012) Genome-wide association analysis of coffee drinking suggests association with CYP1A1/CYP1A2 and NRCAM. Mol Psychiatry 17: 1116–1129. doi: 10.1038/mp.2011.101 21876539 10.1038/mp.2011.101 PMC3482684

[8] Almasy L, Blangero J (1998) Multipoint quantitative-trait linkage analysis in general pedigrees. Am J Hum Genet 62: 1198–1211. doi: 10.1086/301844 9545414 10.1086/301844 PMC1377101

[9] Willer CJ, Li Y, Abecasis GR (2010) METAL: fast and efficient meta-analysis of genomewide association scans. Bioinformatics (Oxford, England) 26: 2190–2191. 10.1093/bioinformatics/btq340 PMC2922887 20616382

[10] Lemaitre RN, Tanaka T, Tang W, Manichaikul A, Foy M, Kabagambe EK, et al (2011) Genetic loci associated with plasma phospholipid n-3 fatty acids: a meta-analysis of genome-wide association studies from the CHARGE Consortium. PLoS genetics 7: e1002193 doi: 10.1371/journal.pgen.1002193 21829377 10.1371/journal.pgen.1002193 PMC3145614

[11] Lemaitre RN, Tanaka T, Tang W, Manichaikul A, Foy M, Kabagambe EK, et al (2011) Genetic loci associated with plasma phospholipid n-3 fatty acids: a meta-analysis of genome-wide association studies from the CHARGE Consortium. PLoS Genet 7: e1002193 doi: 10.1371/journal.pgen.1002193 21829377 10.1371/journal.pgen.1002193 PMC3145614

[12] Katoh M (2004) Human FOX gene family (Review). Int J Oncol 25: 1495–1500. 15492844

[13] Zaitlen N, Kraft P (2012) Heritability in the genome-wide association era. Hum Genet 131: 1655–1664. doi: 10.1007/s00439-012-1199-6 22821350 10.1007/s00439-012-1199-6 PMC3432754

[14] Willett W (1998) Nutritional Epidemiology. New York: Oxford University Press.

[15] Salvini S, Hunter DJ, Sampson L, Stampfer MJ, Colditz GA, Rosner B, et al (1989) Food-based validation of a dietary questionnaire: the effects of week-to-week variation in food consumption. Int J Epidemiol 18: 858–867. 2621022 10.1093/ije/18.4.858

[16] Kaaks R, Slimani N, Riboli E (1997) Pilot phase studies on the accuracy of dietary intake measurements in the EPIC project: overall evaluation of results. European Prospective Investigation into Cancer and Nutrition. Int J Epidemiol 26 Suppl 1: S26–36. 9126531 10.1093/ije/26.suppl_1.s26

[17] Mozaffarian D, Lemaitre RN, Kuller LH, Burke GL, Tracy RP, Siscovick DS (2003) Cardiac benefits of fish consumption may depend on the type of fish meal consumed: the Cardiovascular Health Study. Circulation 107: 1372–1377. 12642356 10.1161/01.cir.0000055315.79177.16

[18] Serra-Majem L, Nissensohn M, Overby NC, Fekete K (2012) Dietary methods and biomarkers of omega 3 fatty acids: a systematic review. Br J Nutr 107 Suppl 2: S64–76. 22591904 10.1017/S000711451200147X

