# Supplementary figures and images for: Genome-wide association meta-analysis of fish and EPA+DHA consumption in 17 US and European cohorts
Source: PLoS One. 2017 Dec 13;12(12):e0186456. doi: 10.1371/journal.pone.0186456 (PMC5728559; doi:10.1371/journal.pone.0186456)

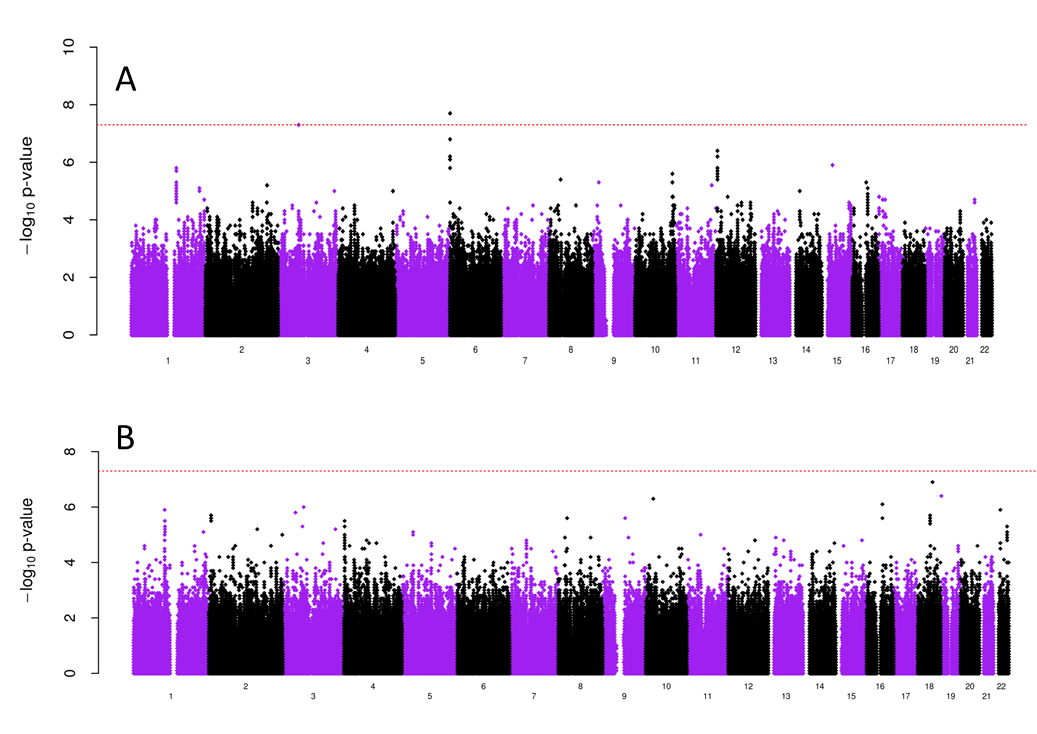

Supplement: S2 Fig — Genome-wide association meta-analysis results of fish (A) and eicosapentanoic acid and docasahexanoic acid (B) with ~2.5 million SNPs graphed by chromosome position and–log10 p-value. (TIFF) [file pone.0186456.s002.tiff]

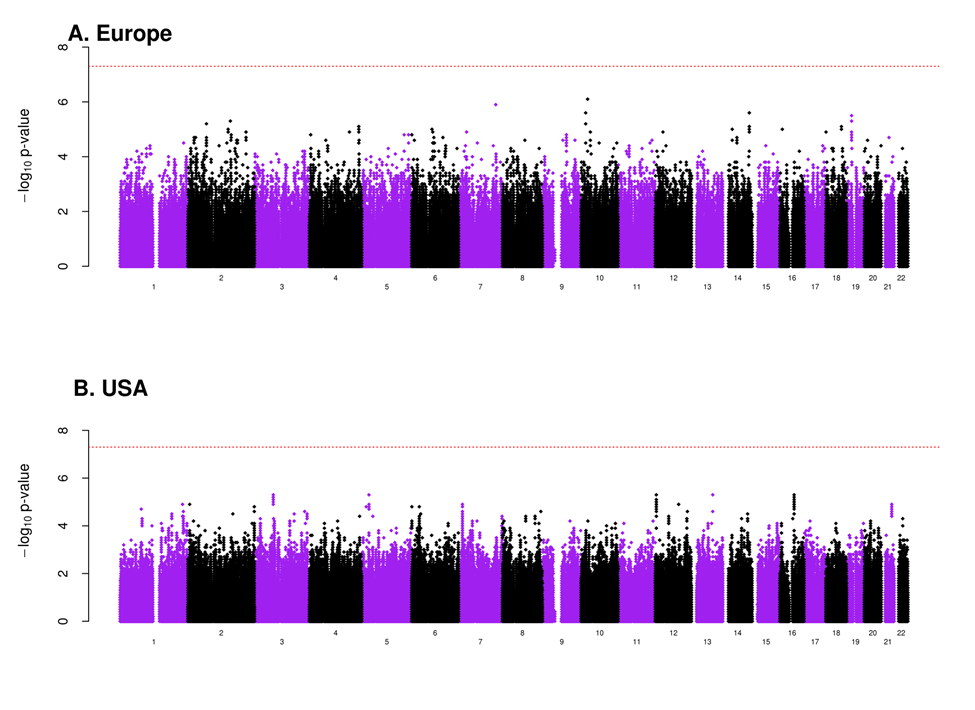

Supplement: S3 Fig — Genome-wide association meta-analysis results of fish consumption in European cohorts (A) and cohorts from the United States (B) for ~2.5 million SNPs graphed by chromosome position and–log10 p-value. (TIFF) [file pone.0186456.s003.tiff]

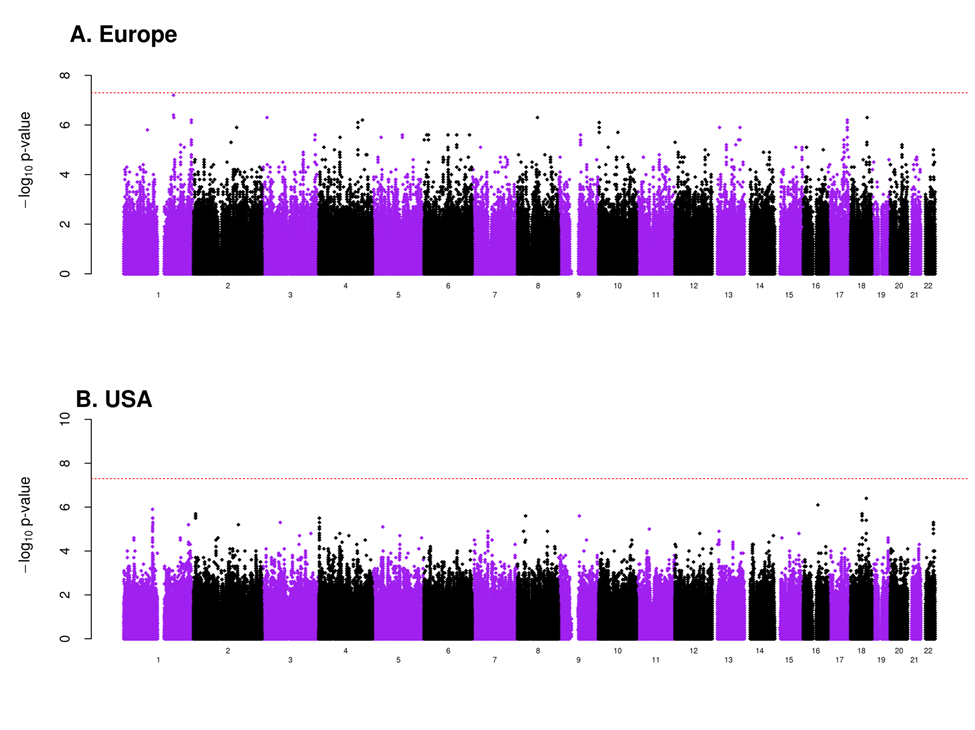

Supplement: S4 Fig — Genome-wide association meta-analysis results of EPA+DHA consumption in European cohorts (A) and cohorts from the United States (B) for ~2.5 million SNPs graphed by chromosome position and–log10 p-value. (TIFF) [file pone.0186456.s004.tiff]
