# Supplementary material for: Genome-wide association meta-analysis of fish and EPA+DHA consumption in 17 US and European cohorts
Source: PLoS One. 2017 Dec 13;12(12):e0186456. doi: 10.1371/journal.pone.0186456 (PMC5728559; doi:10.1371/journal.pone.0186456)
Supplement: S1 Table — (DOCX) [file pone.0186456.s005.docx]

**S1 Table**. Description of cohorts from the CHARGE consortium.

| **Cohort** | **Study description ^1^** | **Web Link and Relevant Reference** |
| --- | --- | --- |
| Atherosclerosis Risk in Communities (ARIC)  USA | The ARIC study is a population-based cohort study designed to study of new and established risk factors for atherosclerosis and community trends in coronary heart disease. In 1987-89, baseline data was collected on 15,792 adults, aged 45–64 y, living in four U.S. communities (Forsyth County, NC; Jackson, MI; northwest Minneapolis suburbs, MN; Washington County, MD). The baseline exam was conducted in 1987-89 and information was collected on African Americans, Whites, and a few adults of other ethnicities, aged 45–64 y. After providing informed consent, 15,792 adults were enrolled (8,710 women and 7,082 men). A total of 7,201, Caucasian adults with available DNA, valid dietary information, and consent to share genetic data were eligible for the current analysis. | [http://www.cscc.unc.edu/aric/  Am J Epidemiol. 129(4): 687-702, 1989 (4)](http://www.cscc.unc.edu/aric/Am%20J%20Epidemiol.%20129(4):%20687-702,%201989%20(4)) |
| Cardiovascular Health Study (CHS)  USA | The CHS is a prospective population-based cohort study of people ≥ 65 years old at baseline initiated to evaluate risk factors for the development and progression of cardiovascular disease. Participants were recruited at four field centers (Forsyth County, NC; Sacramento County, CA; Washington County, MD; Pittsburgh, PA)]. The baseline exam was conducted in 1989-1990. Overall, 5,201 individuals were recruited from random samples of Medicare eligibility lists. A total of 2,765 adults with available DNA, valid dietary information, and consent to share genetic data were eligible for the current analysis. | http://www.chs-nhlbi.org/ Ann Epidemiol. 1(3): 263-276, 1991 (1) |
| Dietary, Lifestyle, and Genetic determinants of Obesity and Metabolic syndrome (DILGOM). Finland | The National FINRISK 2007 Study was carried out in year 2007 in five geographical areas in Finland: the cities of Helsinki and Vantaa (the metropolitan area), the areas of Turku and Loimaa, and the provinces of North Savo, North Karelia, and Oulu. The sample of 10000 men and women aged 25-74 years was a random sample from the Finnish Population Information System, stratified according to sex, 10-year age groups, and the five geographical areas. The survey protocol followed standardized international protocols (Tolonen et al.). Survey included a self-administered questionnaire on socio-demographic factors, health behavior, and medical history, and a health examination, where anthropometric measurements, blood pressure measurements and blood sampling were carried out. The participants who took part in the first phase of the National FINRISK 2007 Study were invited to a more detailed examination on the Dietary, Lifestyle and Genetic Determinants of Obesity and Metabolic syndrome Study (the DILGOM Study) conducted between April and June in 2007. The DILGOM participants have been carefully assessed for their diet (a self-administered food frequency questionnaire (FFQ)), physical activity, psychosocial factors, markers of obesity and glucose metabolism. For the present study, valid dietary and genetic data was available for 604 (GWA genotype data) and 3467 (CardioMetabochip genotype data) individuals. | Konttinen H, Männistö S, Sarlio-Lähteenkorva S, Silventoinen K, Haukkala A. Appetite. 2010 Jun;54(3):473-9.   Inouye M, Silander K, Hämäläinen E, Salomaa V, Harald K, Jousilahti P, Männistö S, Eriksson JG, Saarela J, Ripatti S, Perola M, van Ommen GJ, Taskinen MR, Palotie A, Dermitzakis ET, Peltonen L. PLoS Genet. 2010 Sep 9;6(9).   Peltonen, M., Harald, K., Männistö, S., Saarikoski, L., Peltomäki, P., Lund, L., et al. (2008). The National FINRISK 2007 Study. Helsinki: Publications of the National Public Health Institute, B 34/2008. Available in http://www.ktl.fi/attachments/finriski/2008b34.pdf |
| Estonian Biobank | The Estonian Biobank cohort is a volunteer-based sample of the Estonian resident adult population (aged ≥18 years). At baseline, the general practitioners and medical personnel performed a standardised health examination of the participants, who also donated blood samples for DNA, white blood cells and plasma tests and filled out a 16-module questionnaire on health-related topics such as lifestyle, diet and clinical diagnoses described in WHO ICD-10. | Nelis M. et al. Genetic Structure of Europeans: A View from the North–East. PLoS ONE (2009) 4(5): e5472.  Leitsalu L, Haller T, Esko T, Tammesoo ML, Alavere H, Snieder H, Perola M, Ng PC, Mägi R, Milani L, Fischer K, Metspalu A. Cohort Profile: Estonian Biobank of the Estonian Genome Center, University of Tartu. Int J Epidemiol. (2014) Feb 11. |
| Family Heart Study (FamHS)  USA | The FHS began in 1992 with the ascertainment of 1,200 families (50% randomly sampled, and 50% high risk for CHD). The families (~6,000 individuals,) were sampled on the basis of information on probands from four population-based parent studies: the Framingham Heart Study, the Utah Family Tree Study, and two ARIC centers (Minneapolis, and Forsyth County, NC). Approximately eight years later, study participants belonging to the largest pedigrees were invited for a second clinical exam. A total of 2,767 participants of European descent in 510 extended families were examined. A total of 2,094 adults with available DNA and who provided valid dietary information were eligible for the current study. | [https://dsgweb.wustl.edu/PROJECTS/MP1.html  Higgins et al. Am J Epidemiol. 143 (12): 1219, 1996 (5)](https://dsgweb.wustl.edu/PROJECTS/MP1.htmlHiggins%20et%20al.%20%20Am%20J%20Epidemiol.%20143%20(12):%201219,%201996%20(5)) |
| Framingham Heart Study (FHS)  USA | The Framingham Offspring Study is a community-based longitudinal study designed to examine CVD risk in the offspring of the original participants and their spouses of the Framingham Heart Study cohort. In 1971, 5,124 individuals were enrolled in the study; since then, the cohort has been examined every 3–4 y. Between 1991 and 1995, during the 5th examination cycle, 3,799 adults, with a mean age of 54.98, underwent a standardized medical history and physical examination. Beginning in 2002, 4,095 Gen III participants, who had at least one parent in the offspring cohort, were enrolled in the Framingham Heart Study. At the first cycle of the Gen III study, 4,095 individuals with a mean age of 40 y, underwent the standard clinic examination. For the present study both cohorts were combined for the analysis. A total of 5,835 adults with available DNA, valid dietary information, and consent to share genetic data were eligible for the current study. | [http://www.framinghamheartstudy.org/  Prev Med.4:518–25, 1975 (2) Am J Epidemiol. 165(11):1328-35, 2007 (3)](http://www.framinghamheartstudy.org/Prev%20Med.4:518–25,%201975%20(2)Am%20J%20Epidemiol.%20165(11):1328-35,%202007%20(3)) |
| Health, Aging and Body Composition  (Health ABC).  USA | The Health ABC study is a prospective cohort study investigating the associations between body composition, weight-related health conditions, and incident functional limitation in older adults. Health ABC enrolled well-functioning, community-dwelling black (n=1281) and white (n=1794) men and women aged 70-79 years between April 1997 and June 1998. Participants were recruited from a random sample of white and all black Medicare eligible residents in the Pittsburgh, PA, and Memphis, TN, metropolitan areas. Participants have undergone annual exams and semi-annual phone interviews. A total of 1,249 Caucasian participants who attended the second exam in 1998-1999, and who had available genotyping and food frequency data were eligible for the current study. | <http://www.nia.nih.gov/ResearchInformation/ScientificResources/HealthABCDescription.htm> |
| Health Professionals Follow-up Study | The HPFS was initiated in 1986 when 51,529 male health professionals between 40 and 75 years of age years and residing in the U.S. completed an FFQ and a questionnaire on lifestyle and medical history. The participants have been followed with repeated questionnaires on lifestyle and health every 2 years and FFQs every 4 years. Participants for the current study were those with information on fish consumption and genome-wide scan data. |  |
| Helsinki Birth Cohort Study (HBCS) | HBCS: Helsinki Birth Cohort Study (HBCS) includes 8,760 subjects born in Helsinki between 1934 and 1944. Between 2000 and 2003, a representative subset of 928 males and 1,075 females participated in a clinical study focusing upon cardiovascular and metabolic outcomes, cognitive function and psychological outcomes. A total of 1302 subjects with available DNA, valid dietary information, and consent to share genetic data were eligible for the current study. | Barker DJ, Osmond C, Forsén TJ, Kajantie E, Eriksson JG. Trajectories of growth among children who have coronary events as adults. N Engl J Med. 2005 Oct 27;353(17):1802-9 |
| Health 2000 Study | The Health 2000 survey (H2000), originally designed to provide information on health of the Finnish population, was a nationally representative sample of Finns aged 30 or over and it was carried out from fall 2000 to spring 2001. We obtained a subset of the survey participants for our genetic study by identifying all those who fulfilled the IDF definition of metabolic syndrome criteria, and selecting a matched control for each (previously described by Pajunen et al). A total of 1935 individuals with available genotype data and valid dietary information were eligible for the current study. | http://www.terveys2000.fi/indexe.html   HEALTH AND FUNCTIONAL CAPACITY IN FINLAND, Baseline Results of the Health 2000 Health Examination Survey. In: Aromaa A, Koskinen S, eds. Vol KTL B12/2004. Helsinki: National Public Health Institute; 2004.    Pajunen P, Rissanen H, Härkänen T, Jula A, Reunanen A, Salomaa V. Diabetes Metab. 2010;36:395-401. |
| Invecchiare in Chianti (aging in the Chianti area, InCHIANTI)  Italy | InCHIANTI is a population-based study designed to evaluate the factors that influence mobility in older people in the Chianti region of Tuscany, Italy. A total of 1,616 residents were selected from the population registry of Greve (a rural area: 11,709 residents with 19.3% of the population greater than 65 years of age), and Bagno a Ripoli (Antella village near Florence; 4,704 inhabitants, with 20.3% greater than 65 years of age). The participation rate was 90% (n=1453), and the participants ranged between 21-102 years of age. For the present study, 1,071 adults with available DNA and who provided complete dietary information were eligible for the current study. | [http://www.inchiantistudy.net/bindex.html  Ferrucci L, et al. J Am Geriatr Soc. 48:1618-1625, 2000 (12)](http://www.inchiantistudy.net/bindex.htmlFerrucci%20L,%20et%20al.%20J%20Am%20Geriatr%20Soc.%2048:1618-1625,%202000%20(12)) |
| Multi-Ethnic Study of Atherosclerosis (MESA) | The MESA is a cohort study designed to investigate the characteristics of subclinical cardiovascular disease and the risk factors that predict progression to clinically overt cardiovascular disease or progression of the subclinical disease. MESA comprises a diverse, population-based sample of 6,814 asymptomatic men and women aged 45-84. Thirty-eight percent of the recruited participants are Caucasian, 28 percent African-American, 22 percent Hispanic, and 12 percent Asian, predominantly of Chinese descent. Participants were recruited from six field centers across the United States: Wake Forest University, Columbia University, Johns Hopkins University, University of Minnesota, Northwestern University and University of California - Los Angeles. In the current analysis only data from Caucasian participants were analyzed, including up to 2302 unrelated adults with valid dietary information and consent to share genetic data. | [http://www.mesa-nhlbi.org/  Bild DE, Bluemke DA, Burke GL, Detrano R, Diez Roux AV, Folsom AR, Greenland P, Jacob DR,Jr, Kronmal R, et al. Multi-ethnic study of atherosclerosis: Objectives and design. Am J Epidemiol. 2002 Nov 1;156(9):871-81.](http://www.mesa-nhlbi.org/Bild%20DE,%20Bluemke%20DA,%20Burke%20GL,%20Detrano%20R,%20Diez%20Roux%20AV,%20Folsom%20AR,%20Greenland%20P,%20Jacob%20DR,Jr,%20Kronmal%20R,%20et%20al.%20Multi-ethnic%20study%20of%20atherosclerosis:%20Objectives%20and%20design.%20Am%20J%20Epidemiol.%202002%20Nov%201;156(9):871-81.) |
| Nurse's Health Study | The NHS was established in 1976 when 121,700 female registered nurses aged 30-55 years and residing in 11 large U.S. states completed a mailed questionnaire on medical history and lifestyle characteristics (PMID 15864280). Every two years, follow-up questionnaires have been sent to update information on exposures and newly diagnosed diseases and every 2 to 4 years diet was assessed using a validated semi-quantitative FFQ (Willett, Nutritional Epidemiology). Participants for the current study were those with information on fish consumption and genome-wide scan data. |  |
| Rotterdam Study Netherlands | The Rotterdam Study a prospective cohort study among, initially, 7,983 persons living in Rotterdam in The Netherlands (78% of 10,215 invitees). The first cohort (RS-I) started in 1990 in Ommoord, a suburb of Rotterdam, the Netherlands, comprising of 7,983 men and women aged 55 years and over. Baseline measurements were obtained between 1990 and 1993. Trained research assistants collected data on current health status, use of medication, medical history, lifestyle and risk indicators for chronic diseases during an extensive home interview. Subsequently, the participants visited the study center for detailed clinical examinations. Follow up visits were held every 2-3 years. | Hofman, Albert, Sarwa Darwish Murad, Cornelia M. van Duijn, Oscar H. Franco, André Goedegebure, M. Arfan Ikram, Caroline C. W. Klaver, et al. “The Rotterdam Study: 2014 Objectives and Design Update.” Eur J Epidemiol (2013) 28:889–926  http://www.erasmus-epidemiology.nl/research/ergo.htm |
| The Hellenic Study of Interactions between SNPs and Eating in Atherosclerosis Susceptibility (THESIAS) | The Hellenic Study of Interactions between Snps and Eating in Atherosclerosis Susceptibility (THISEAS) study is a case- control study designed to investigate the association between genetic and lifestyle environmental factors and the risk of coronary artery disease in men and women aged >25 yrs. The control group consists of individuals with no history of cardiovascular disease, while cases are individuals with coronary artery disease. Hematological, biochemical and anthropometric measurements were conducted to all participants. Dietary assessment and physical activity data were collected through face to face interview by well trained scientists. Exclusion criteria for the control group were history of cardiovascular disease, cancer and/ or other inflammatory disease. The population for the present analysis was comprised of 395 subjects with phenotype, genotype and dietary data available. | PMID:20167083 |
| Women's Genome Health Study | The Women’s Genome Health Study (WGHS) is a prospective cohort of initially healthy, female North American health care professionals at least 45 years old at baseline representing participants in the Women’s Health Study (WHS) who provided a blood sample at baseline and consent for blood-based analyses. The WHS was a 2x2 trial beginning in 1992-1994 of vitamin E and low dose aspirin in prevention of cancer and cardiovascular disease with about 10 years of follow-up. Since the end of the trial, follow-up has continued in observational mode. Additional information related to health and lifestyle were collected by questionnaire throughout the WHS trial and continuing observational follow-up. | Ridker PM, Chasman DI, Zee RY, Parker A, Rose L, Cook NR, Buring JE; Women's Genome Health Study Working Group. Rationale, design, and methodology of the Women's Genome Health Study: a genome-wide association study of more than 25,000 initially healthy american women. Clin Chem. 2008 Feb;54(2):249-55.PMID: 18070814 |
| YFS | The Cardiovascular Risk in Young Finns (YFS) is a population-based 27 year follow up-study. The first cross-sectional survey was conducted in 1980, when 3,596 Caucasian subjects aged 3-18 years participated. In adulthood, the 27-year follow-up study was conducted in 2007 (ages 30-45 years) with 2,204 participants. The study cohort for the present analysis comprised subjects who had participated in the study in 2007 and had validated dietary data from FFQ, available genotype and other risk factor data. The study was approved by the local Ethical Committees and was performed according to Helsinki declaration. | [http://youngfinnsstudy.utu.fi/ Raitakari OT et al. Cohort profile. Int. J Epidemiol. 2008;37:1220-6](http://youngfinnsstudy.utu.fi/Raitakari%20OT%20et%20al.%20Cohort%20profile.%20Int.%20J%20Epidemiol.%202008;37:1220-6) |
