# Supplementary material for: Genome-wide association meta-analysis of fish and EPA+DHA consumption in 17 US and European cohorts
Source: PLoS One. 2017 Dec 13;12(12):e0186456. doi: 10.1371/journal.pone.0186456 (PMC5728559; doi:10.1371/journal.pone.0186456)
Supplement: S2 Table — (DOCX) [file pone.0186456.s006.docx]

**S2 Table**. Dietary assessment methods for CHARGE cohorts.

| **Study** | **Description** | **FFQ Line items / Top contributing Food Groups** |
| --- | --- | --- |
| Atherosclerosis Risk in Communities (ARIC) USA | An interviewer-administered, 66-item semi-quantitative FFQ that was modified from the validated Willett 61-item FFQ (19) (modifications described elsewhere (20)). Participants were asked to indicate how often, on average, they consumed various foods and beverages over the past year according to 9 frequency categories, ranging from never or <1 time/mo to ≥6 times/d. Standard portion sizes given as a reference for intake estimation. Supplementary questions included regarding frequency of fried food consumption and brand name of the breakfast cereal most commonly consumed (open-ended response). Dietary information was judged as unreliable and excluded from further analysis if total energy intake was estimated to be <500 or >3600 kcal for women and <600 or >4200 kcal for men or if 10 or more items of the FFQ were unanswered.  Related References: Willett WC, et al. Am J Epidemiol. Jul 1985;122(1):51-65. (19) Stevens J et al. Nutrition Research 1996;16: 735-745. (20) | 4 items:  canned tuna fish; dark meat fish; other fish; shrimp, lobster, scallops |
| Cardiovascular Health Study (CHS) USA | Usual dietary intake was assessed using a picture-sort version of the National Cancer Institute FFQ. This is a 99-item, self-administered FFQ. Participants were asked to indicate how often, on average, they consumed various foods and beverages over the past year according to 9 frequency categories, ranging from never to >5 times per week.. Portion sizes were illustrated by color pictures or laminated 4 X 6 in (10 X 15 cm) index card with a black-and white line drawing. Dietary information was judged as unreliable and excluded from further analysis if calculated total kilocalories were < 500 or > 5000 kcal/d. Related References: Kumanyika S, et al. J Am Diet Assoc. 1996 Feb;96(2):137-44. (16) | 2 line items: 1) tuna fish/tuna salad/tuna casserole and 2) other fish, broiled or baked. |
| Dietary, Lifestyle, and Genetic Determinants of Obesity and Metabolic Syndrome (DILGOM) | Food consumption over the previous 12 months was assessed with a validated self-administered FFQ updated for this study. The average use of 132 food items and mixed dishes were recorded by nine frequency categories ranged from never or seldom to at least six times a day. The portion size was fixed for each food item and mixed dish (e.g., slice and glass). Reporting additional items consumed frequently but not listed in the FFQ was also allowed. The participants completed the FFQ at the study site, where a trained study nurse reviewed the questionnaire. Exclusions were made due to incompletely filled FFQs (n = 74) and daily energy intake cut-offs corresponding to 0.5 % at both ends of the daily energy intake distributions for men and women (n = 48). The average daily intakes of food groups and nutrients were calculated by the national food composition database, Fineli (http://www.fineli.fi/index.php), using in-house software. References: Männistö S, Virtanen M, Mikkonen T, Pietinen P. Reproducibility and validity of a food frequency questionnaire in a case-control study on breast cancer. J Clin Epidemiol 1996;49:401-409. Paalanen L, Männistö S, Virtanen MJ, Knekt P, Räsänen L, Montonen J, Pietinen P. Validity of a food frequency questionnaire varied by age and body mass index. J Clin Epidemiol. 2006 Sep;59(9):994-1001 Reinivuo H, Hirvonen T, Ovaskainen ML, Korhonen T, Valsta LM. Dietary survey methodology of FINDIET 2007 with a risk assessment perspective. Public Health Nutr 2010;13:915-919. | Fish soup Frozen fish or fish fingers Salmon or rainbow trout Baltic herring Pikeperch, whitefish, perch, vendace or pike Smoked fish (e.g. whitefish, salmon or rainbow trout) Spiced or salted fish Tuna or other canned fish Kalakukko [rye bread fish pasty] Shrimp or crayfish |
| Estonian Biobank | A questionnaire administered by trained interviewers. Participants were asked to indicate how often, on average, they consumed various foods and beverages over the past year according to 4 frequency categories: never; 1-2 times/week; 3-5 times/week, ≥6 times/week. |  |
| Family Heart Study (FamHS) USA | A 66-item questionnaire modified from the Willet FFQ administered by trained interviewers. Participants were asked to indicate how often, on average, they consumed various foods and beverages over the past year according to 9 frequency categories, ranging from never or <1 time/mo to ≥6 times/d. Portion sizes were specified. Dietary information was judged as unreliable and excluded from further analysis if reported energy intakes were <3347.2kJ/day (799.3 kcal/day) or >17572.8 kJ/day (4196.4 kcal/day) for men and <2510.4 kJ/d (599.5 kcal/day) or >14644 kJ/day (3497 kcal/day) for women.  Related References: Stein AD et al. Am J Epidemiol 1992;135(6):667-677. (21) Willett WC, et al. Am J Epidemiol. Jul 1985;122(1):51-65. (19) | 4 items:  canned tuna fish dark meat fish (salmon, mackerel, swordfish, sardines, bluefish) Other fish (cod, perch, catfish, etc) Shrimp, lobster, scallops |
| Framingham Heart Study (FHS) USA | A self-administered 126-item FFQ. Participants were asked to indicate how often, on average, they consumed various foods and beverages over the past year according to 9 frequency categories, ranging from never or <1 time/mo to ≥6 times/d. Portion sizes were specified. Separate questions about the use of vitamin and mineral supplements and the type of breakfast cereal most commonly consumed were also included in the FFQ. Dietary information was judged as unreliable and excluded from further analysis if reported energy intakes were < 2.51 MJ/d (600 kcal/d) or > 16.74 MJ/d (4000 kcal/d) for women and > 17.57 MJ/d (4200 kcal/d) for men or if >= 12 food items were left blank.  Related References: Rimm et al. Am J Epidemiol 1992;135:1114–26, 1127–36. (17) Salvini S et al. Int J Epidemiol 1989;18:858–67. (18) | 4 items:  1. canned tuna fish 2. dark meat fish e.g. mackerel, salmon, sardines, bluefish, swordfish) 3. other fish 4. shrimp, lobster, scallops as a main dish |
| Helsinki Birth Cohort Study (HBCS) | Diet was assessed with a validated, self-administered 128-item FFQ. The FFQ was designed to assess the ordinary diet over the previous 12 mo. The subjects were asked to indicate the average intake frequency of each food item and mixed dish. The 9 possible frequency categories ranged from never or seldom to ≥6 times/d. The portion sizes were fixed, eg, a glass or a slice of bread. Food and nutrient intake was calculated using the Finnish Food Composition Database, Fineli. Dietary information was judged as unreliable and excluded from further analysis if reported energy intakes were <650 or >6100 kcal/d, corresponding to 0.5% at each end of the selfreported daily energy intake scale or if ≥12 food items were left blank. Related References: Männistö et al. J Clin Epidemiol 1996;49:401-9.  Paalanen et al. J Clin Epidemiol 2006;59:994-1001. | Fish soup Frozen fish or fish fingers Salmon or rainbow trout Baltic herring Pikeperch, whitefish, perch, vendace or pike Spiced or salted fish Tuna or other canned fish Kalakukko (rye bread fish pasty) Shrimp or crayfish |
| Health, Aging and Body Composition (Health ABC). USA | A 108-item interviewer-administered FFQ (Block Dietary Data Systems, Berkeley, CA). Participants were asked to indicate how often, on average, they consumed various foods and beverages over the past year according to nine frequency categories, ranging from “never” to “every day”. Portion size information was collected by trained interviewers using wood blocks, food models, standard kitchen measures, and flash cards to help participants estimate portion sizes. Individuals with serious errors (skipped >15% of items or reported <3 or >20 foods/day) on the FFQ and those who reported energy intakes less than 500 kcal/d or greater than 3,500 kcal/d in women and less than 800 kcal/d or greater than 4,000 kcal/d in men were excluded.  Related References: Houston et al. Am J Clin Nutr. 2008; 87(1):150-5. (15) | 4 items -- 1: shellfish like shrimp, scallops, crab 2: tuna, tuna salad, tuna casserole 3: fried fish or fish sandwich 4: other fish, broiled or baked |
| HPFS | Fish intake was assessed as described for NHS. For the present analysis, we included the participants mean total fish intakes of the 1986 and 1990 FFQs. | 1: canned tuna fish 2: dark meat fish 3: other fish 4: shrimp, lobster, scallops as a main dish |
| H2000 | Dietary data were collected using food frequency questionnaire of the preceding year. It consisted of 128 commonly used or nutrionally important food items and mixed dishes. Validity of this FFQ meets the requirements of epidemiological studies (1, 2). The items were grouped under 12 sub-headings one of them being fish dishes. Nine frequency categories ranged from “never or rarely” to “six or more times per day”. The portion sizes were fixed and if possible, specified using natural units (e.g. serving slice, glass, cup). Fish consumption was converted into grams per day by multiplying the food consumption frequency by fixed portion sizes. Food and nutrient intake was calculated using the Finnish Food Composition Database (http://www.fineli.fi/index.php). Fasting blood samples were collected to measure serum concentrations of fatty acids in a Health 2000 Sub-study (3). Serum fatty acids composition was analyzed using a gas chromatograph (4). The fish-derived long-chain n-3 fatty acids (eicosapentaenoic acid, docosahexaenoic acid and docosapentaenoic acid) were expressed in mg/L and as proportions from total fatty acids. References: 1. Paalanen L, Männistö S, Virtanen MJ, Knekt P, Räsänen L, et al. (2006) Validity of a food frequency questionnaire varied by age and body mass index. J Clin Epidemiol 59: 994–1001. 2. Männistö S, Virtanen M, Mikkonen T, Pietinen P (1996) Reproducibility and validity of a food frequency questionnaire in a case-control study on breast cancer. J Clin Epidemiol 49: 401–409. 3. Suominen-Taipale AL, Partonen T, Turunen AW, Männistö S, Jula A, Verkasalo PK. Fish consumption and omega-3 polyunsaturated fatty acids in relation to depressive episodes: a cross-sectional analysis.PLoS One. 2010 May 7;5(5):e10530. 4. Jula A, Marniemi J, Rönnemaa T, Virtanen A, Huupponen R (2005) Effects of diet and simvastatin on fatty acid composition in hypercholesterolemic men: a randomized controlled trial. Arterioscler Thromb Vasc Biol 25: 1952–1959. | Fish soup Frozen fish or fish fingers Salmon or rainbow trout Baltic herring Pikeperch, whitefish, perch, vendace or pike Spiced or salted fish Tuna or other canned fish Kalakukko (rye bread fish pasty) Shrimp or crayfish |
| Invecchiare in Chianti (aging in the Chianti area, InCHIANTI) Italy | A 236 item, interviewer administered FFQ that investigates how frequently (weekly, monthly, yearly) each specific food was generally consumed. Participant is asked to specify the size of the portion usually consumed, in comparison to a range of portion that are shown in colored photographs. Nutrient data for specific foods were obtained from the Food Composition Database for Epidemiological Studies in Italy (18). Dietary information was judged as unreliable and excluded from further analysis if reported energy intakes less than 600 kcal/d or greater than 4,000 kcal/d and 4,200 kcal/d in women and men, respectively.  Related References: Bartali et al. Arch. Gerontol Geriatr. Geriatr. 38 2004; 51–60. (30) Pisani et al. Int J Epidemiol. 1997; 26:152–160. (31) | salted fish, canned fish, cod, flounder, sardines, trout, sword fish, other kind of fish |
| Multi-Ethnic Study of Atherosclerosis (MESA) | 120-item, self-administered, modified-Block FFQ [Mayer-Davis E et al. Ann Epidemiol 1999;9:314–324. & Nettleton JA et al. Br J Nutr 2009; 102, 1220–1227.] | shrimp, lobster, crab, oysters, mussels (not fried); tuna, salmon, sardines (including sashimi or sushi); other broiled, steamed, baked or raw fish (trout, sole, halibut, poke, grouper); fried fish or fish sandwich, fried shrimp, calamari; fish stew or seafood gumbo, paella; stir-fried shrimp or fish with vegetables; |
| Nurse's Health Study | For the present analysis, we included the participants’ mean total fish intakes of the 1984 and 1986 FFQs. The FFQ included four line items for canned tuna, dark fish, other fish and seafood main dishes. For each item, participants were asked how often, on average, they had consumed a specified amount of each food over the past year. The participants could choose from nine frequency categories (never, 1-3 per month, 1 per week, 2-4 per week, 5-6 per week, 1 per day, 2-3 per day, 4-5 per day and 6 or more per day). We assessed the total number of fish servings/d by summing across line items. | 1: canned tuna fish 2: dark meat fish 3: other fish 4: shrimp, lobster, scallops as a main dish |
| Rotterdam Study Netherlands | Dietary assessment followed a two-step procedure: 1) A simple self-administered questionnaire was first completed at home, only questions were asked about which food items were consumed; no questions about portion sizes (or frequency) were asked during this step. 2) A subsequent structured interview was later conducted at the research center with a trained dietitian Participants were asked to indicate how often, on average, they consumed various foods and beverages over the past year according to 9 frequency categories, ranging from never or <1 time/mo to ≥6 times/d. Portion sizes were presented in natural units (eg. slices of bread) or household measures (e.g., cups, bowls, tablespoons, plates, etc.) Nutritional supplement intakes were not considered because dose and duration were not recorded with sufficient accuracy. Dietary information was judged as unreliable and excluded from further analysis if a dietician considered the reported dietary intake unreliable, i.e. because participant's answers during the dietary interview were either too inconsistent or too incomplete. Related References: Klipstein-Grobusch K, et al. Eur J Clin Nutr. 1998 Aug; 52(8):588-96. (29) | Eel ; Fish lean 0-2 g fat ; Fish medium fat ; Fish fat > 10 g fat ; Herring salted ; Mackerel ; Sardines ; Pilchards in oil canned ; Salmon canned ; Eel smoked ; Plaice ; Fish fingers ; Cod ; Herring marinated |
| The Hellenic Study of Interactions between SNPs and Eating in Atherosclerosis Susceptibility (THESIAS) | Dietary assessment data was collected through face to face interview by well trained scientists. A semi- quantitative 172- item questionnaire was used to assess dietary intake. Participants were asked to indicate how often they consumed various foods and beverages, as well as the portion size by comparison with photos. Daily consumption was calculated from the FFQ by multiplying the standard serving size of each food (as described by the Ministry of Health and Welfare, Supreme Scientific Health Council) by the value corresponding to each consumption frequency: never; 1–3 times/month ; 1–2 times/week; 3–4 times/week; 5-6 times/week; 1time/day. Related References: Ministry of Health and Welfare, Archives of Hellenic Medicine 1999, 16(5): 516-524 | Canned fish (tuna, salmon etc), Small Fatty Fish fresh or frozen (sardine, anchovy etc), Other small fish fresh or frozen (smelts), Big fatty fish fresh or frozen(salmon, mackerel etc), Other big fish fresh or frozen, Seafood |
| Women's Genome Health Study (WGHS) | Dietary omega-3 fatty acids were derived from a previously validated baseline 128-food-frequency questionnaire. Fish consumption was assessed through 4 items on the FFQ. Participants were asked to report their average consumption of canned tuna (3–4 oz), dark-meat fish (3–5 oz), other fish (3–5 oz), and shrimp, lobster, and scallops as a main dish over the past year. Possible responses included never or <1 time/mo, 1–3 times/mo, 1 time/wk, 2–4 times/wk, 5–6 times/wk, 1 time/d; 2–3 times/d, 4–5 times/d, and ≥6 times/d. Individual responses were converted into servings per day by using the midpoint for each response category. The fish variable (servings/day) was created by the summing the frequency of consumption of canned tuna, dark fish, other fish, and shrimp, lobster, and scallops as a main dish. Related references:  Willett WC, Sampson L, Stampfer MJ, Rosner B, Bain C, Witschi J, Hennekens CH, Speizer FE. Reproducibility and validity of a semiquantitative food frequency questionnaire. Am J Epidemiol. 1985 Jul;122(1):51-65. PubMed PMID: 4014201 Djoussé L, Gaziano JM, Buring JE, Lee IM. Dietary omega-3 fatty acids and fish consumption and risk of type 2 diabetes. Am J Clin Nutr. 2011 Jan;93(1):143-50. PMID: 20980491 | 1: canned tuna fish 2: dark meat fish 3: other fish 4: shrimp, lobster, scallops as a main dish |
| Young Finns Study (YFS) | Dietary data were collected using a 131-item food frequency questionnaire, self-administered and checked by a nurse. Participants were asked to report their food consumption during the previous 12 months. The questionnaire had fixed portion sizes and 9 response categories from “never or rarely” to “6 or more times per day”. Related references: 1. Paalanen L, Männistö S, Virtanen MJ, Knekt P, Räsänen L, et al. Validity of a food frequency questionnaire varied by age and body mass index. J Clin Epidemiol 2006;59: 994–1001. 2. Männistö S, Virtanen M, Mikkonen T, Pietinen P. Reproducibility and validity of a food frequency questionnaire in a case-control study on breast cancer. J Clin Epidemiol 1996;49:401–409. | Fish soup Frozen fish or fish fingers Salmon or rainbow trout Baltic herring Pikeperch, whitefish, perch, vendace or pike Spiced or salted fish Tuna or other canned fish Kalakukko (rye bread fish pasty) Shrimp or crayfish |
