# Supplementary material for: Genome-wide association meta-analysis of fish and EPA+DHA consumption in 17 US and European cohorts
Source: PLoS One. 2017 Dec 13;12(12):e0186456. doi: 10.1371/journal.pone.0186456 (PMC5728559; doi:10.1371/journal.pone.0186456)
Supplement: S3 Table — (DOCX) [file pone.0186456.s007.docx]

**S3 Table3.** Genotyping Methods for CHARGE cohorts

|  |  | **ARIC** | **CHS** | **DILGOM** | **Estonian Study** | **FamHS** |
| --- | --- | --- | --- | --- | --- | --- |
|  | Array | Affymetrix 6.0 | Illumina HumanCNV370-Duo BeadChip | Illumina BeadChip Human 610-Quad | Illumina 370CNV, Illumina OmniExpress | Illumina 510, 650, and 1M |
|  | Genotype Calling software | Birdseed | Illumina BeadStudio | Illuminus | Illumina BeadStudio | Illumina BeadStudio Suite |
|  | Sample Size | 9557 | 3230 | 604 | 9920 | 3523 |
| Genotyping QC | Exlusions (subject): |  |  |  |  |  |
|  | Call rate | < 95% | < 95% | < 95% | < 95% | < 98% |
|  | other | sex mismatch, first-degree relative, genetic outlier | duplicate, Mendelian inconsistensies, heterozygote frequency=0 | heterozygosity, gender check and relatedness checks | heterozygosity, gender check and relatedness checks | Mendelian inconsistencies |
|  | Exlusions (SNP): |  |  |  |  |  |
|  | Call rate | < 95% | < 95% | < 95% | < 98% | < 98% |
|  | MAF | < 1% | n.a. | < 1% | < 1% | < 1% |
|  | HWE | < 10^-5^ | < 10^-5^ | NA | < 10^-6^ | < 10^-6^ |
| Imputation information | Imputation program | MACH | BIMBAM10 v0.91 | MACH 1.0 (Hapmap r22 CEU) | IMPUTE | MACH (v 1.0.16) |
|  | NCBI build for Imputation | 36 | 35 | 36 | 36 | 36 |
|  | analysis program | ProbABLE | R | ProbABEL | ProbABEL | SAS, R |

**S3 Table, cont’d.** Genotyping Methods for CHARGE cohorts

|  |  | **FHS** | **Health ABC** | **HPFS_T2D** | **HPFS_CHD** | **HPFS_KS** |
| --- | --- | --- | --- | --- | --- | --- |
|  | Array | Affymetrix 500K and MIPS 50K | Illumina Human 1M-Duo BeadChip | Affymetrix 6.0 | Affymetrix 6.0 | Illumina 610Q |
|  | Genotype Calling software | BRLMM | Birdseed | BirdSeed | BirdSeed | Beadstudio |
|  | Sample Size | 6371 | 1497 | 2456 | 1133 | 547 |
| Genotyping QC | Exlusions (subject): |  |  |  |  |  |
|  | Call rate | < 97% | <97% | <=98% | <=98% | < 95% |
|  | other | Heterozygosity filter 5SD from eman, > 1000 Mendelian errors | sample failure, sex mismatch, first-degree relative | -sex discrepancy with genetic data from X-linked markers -duplicates and first/second degree relatives -PCA outliers -heterozygosity -autosomal chromosome abberations -missing phenotype & covariate information | -duplicates and first/second degree relatives -PCA outliers -heterozygosity -missing phenotype & covariate information | -duplicates and first/second degree relatives -PCA outliers -missing phenotype & covariate information |
|  | Exlusions (SNP): |  |  |  |  |  |
|  | Call rate | < 95% | < 97% | <=98% | <=98% | < 95% |
|  | MAF | < 1% | < 1% | < 2% | < 2% | < 1% |
|  | HWE | < 10^-6^ | < 10^-6^ | <1e-4 | <1e-4 | <1e-5 |
| Imputation information | Imputation program | MACH | MACH 1.0.16 | MACH | MACH | MACH |
|  | NCBI build for Imputation | release 22,build 36 | 36 | 36 | 36 | 36 |
|  | analysis program | R | R | ProbABLE | ProbABLE | ProbABLE |

**S3 Table, cont’d.** Genotyping Methods for CHARGE cohorts

|  |  | **HBCS** | **H2000** | **InCHIANTI** | **MESA** | **NHS_T2D** |
| --- | --- | --- | --- | --- | --- | --- |
|  | Array | modified Illumina 610k | Illumina Human610-Quad BeadChip | Illumin 550K | Affymetrix 6.0 | Affymetrix 6.0 |
|  | Genotype Calling software | Illumina BeadStudio | Illuminus | Beadstudio | Birdseed | BirdSeed |
|  | Sample Size | 1701 | 1935 | 1194 | 2302 | 3145 |
| Genotyping QC | Exlusions (subject): |  |  |  |  |  |
|  | Call rate | < 95% | < 95% | < 98.5 | < 95% | <=98% |
|  | other | sex mismatch, first-degree relative, genetic outlier | heterozygosity, gender check and relatedness checks | sex mispecifications, heterozygosity > 0.3, duplicate | Heterozygosity < 53% | -sex discrepancy with genetic data from X-linked markers -duplicates and first/second degree relatives -PCA outliers -heterozygosity -autosomal chromosome abberations -missing phenotype & covariate information |
|  | Exlusions (SNP): |  |  |  |  |  |
|  | Call rate | < 95% | < 95% | < 99 | < 95% | <=98% |
|  | MAF | < 1% | < 5% | <1% | NA | < 2% |
|  | HWE | < 10^-6^ | NA | <10^-4^ | NA | <1e-4 |
| Imputation information | Imputation program | MACH | MACH 1.0 (Hapmap r22 CEU) | MACH 1.0.16 | IMPUTE (2.1.0) | MACH |
|  | NCBI build for Imputation | release 22,build 36 | 36 | 36 | 36 | 36 |
|  | analysis program | Plink/ ProbABEL | ProbABEL | MERLIN | Plink/SNPTEST | ProbABLE |

**S3 Table, cont’d.** Genotyping Methods for CHARGE cohorts

|  |  | **NHS_CHD** | **NHS_KS** | **NHS_Cancer** | **Rotterdam** | **THISEAS** |
| --- | --- | --- | --- | --- | --- | --- |
|  | Array | Affymetrix 6.0 | Illumina 610Q | Illumina 550 | Illumina 550K | OmniExpress |
|  | Genotype Calling software | BirdSeed | Beadstudio | Beadstudio | BeadStudio | Illuminus |
|  | Sample Size | 1102 | 478 | 2054 | 4606 | 1075 |
| Genotyping QC | Exlusions (subject): |  |  |  |  |  |
|  | Call rate | <=98% | < 95% | <=90% | < 97.5% | <95% |
|  | other | -duplicates and first/second degree relatives -PCA outliers -heterozygosity -missing phenotype & covariate information | -duplicates and first/second degree relatives -PCA outliers -missing phenotype & covariate information | -duplicates and first/second degree relatives -PCA outliers -missing phenotype & covariate information | excess autosomal heterozygosity, sex mismatch, genetic outlier | duplicates, heterozygosity, ethnic outliers, sex mismatch |
|  | Exlusions (SNP): |  |  |  |  |  |
|  | Call rate | <=98% | < 95% | <=90% | < 90% | <98% |
|  | MAF | < 2% | < 1% | < 1% | < 1% | na |
|  | HWE | <1e-4 | <1e-5 | n.a. | < 10^-6^ | <10-4 |
| Imputation information | Imputation program | MACH | MACH | MACH | MACH | na |
|  | NCBI build for Imputation | 36 | 36 | 36 | 36 | na |
|  | analysis program | ProbABLE | ProbABLE | ProbABLE | ProbABEL | Plink |

**S3 Table, cont’d.** Genotyping Methods for CHARGE cohorts

|  |  | **WGHS** | **Young Finns** | **DILGOM (Metabochip)** |
| --- | --- | --- | --- | --- |
|  | Array | Illumina HumanHap300 Duo+ | Illumina 670K | Illumina Metabochip |
|  | Genotype Calling software | BeadStudio v3.3 | Illuminus | GenCall |
|  | Sample Size | 23294 | 1815 | 3467 |
| Genotyping QC | Exlusions (subject): |  |  |  |
|  | Call rate | <98% | < 95% | < 95% |
|  | other | NA | duplicates, heterozygosiy, sex mismatch, relatedness | 1) heterozygosity <23.9% or >27.6%; 2) ethnic outliers;  3) related individuals and duplicates. |
|  | Exlusions (SNP): |  |  |  |
|  | Call rate | <90% | < 95% | < 95% |
|  | MAF | <1% | < 1% | < 1% |
|  | HWE | <10-6 | < 10^-6^ | < 10^-6^ |
| Imputation information | Imputation program | MACH 1.0.16 | MACH 1.0 | NA |
|  | NCBI build for Imputation | 36 | 36 | NA |
|  | analysis program | ProbABEL | ProbABEL | ProbABEL |
