# Supplementary material for: Genome-wide association meta-analysis of fish and EPA+DHA consumption in 17 US and European cohorts
Source: PLoS One. 2017 Dec 13;12(12):e0186456. doi: 10.1371/journal.pone.0186456 (PMC5728559; doi:10.1371/journal.pone.0186456)
Supplement: S4 Table — (DOCX) [file pone.0186456.s008.docx]

**S4 Table.** Associations of top Fish and EPA+DHA SNPs with circulating DHA and EPA levels*

| **SNPID** | **Effect/ Non-effect** | **DHA GWAS results** | | |  | **EPA GWAS results** | | |
| --- | --- | --- | --- | --- | --- | --- | --- | --- |
|  |  | **Effect** | **StdErr** | **P-value** |  | **Effect** | **StdErr** | **P-value** |
| *Top Fish Intake SNPs* | |  |  |  |  |  |  |  |
| rs9502823 | A/G | -0.006 | 0.063 | 0.929 |  | -0.013 | 0.018 | 0.493 |
| rs17396472 | A/T | -0.137 | 0.093 | 0.142 |  | 0.010 | 0.030 | 0.745 |
| rs1860343 | T/C | -0.009 | 0.014 | 0.520 |  | -0.007 | 0.005 | 0.126 |
| rs1562806 | T/C | -0.043 | 0.063 | 0.493 |  | -0.013 | 0.023 | 0.583 |
| rs16834168 | A/G | 0.037 | 0.057 | 0.520 |  | 0.011 | 0.016 | 0.504 |
| *Top EPA+DHA Intake SNPs* | | |  |  |  |  |  |  |
| rs11877506 | A/G | -0.045 | 0.054 | 0.406 |  | 0.009 | 0.019 | 0.652 |
| rs2456163 | T/C | -0.010 | 0.043 | 0.812 |  | -0.020 | 0.013 | 0.110 |
| rs7476409 | T/C | -0.030 | 0.049 | 0.545 |  | -0.004 | 0.015 | 0.775 |
| rs7206790 | C/G | -0.006 | 0.017 | 0.718 |  | -0.001 | 0.006 | 0.938 |

*Results from Lemaitre RN, Tanaka T, Tang W, Manichaikul A, Foy M, et al. (2011) Genetic loci associated with plasma phospholipid n-3 fatty acids: a meta-analysis of genome-wide association studies from the CHARGE Consortium. PLoS Genet 7: e1002193
