# Supplementary material for: Genome-wide association meta-analysis of fish and EPA+DHA consumption in 17 US and European cohorts
Source: PLoS One. 2017 Dec 13;12(12):e0186456. doi: 10.1371/journal.pone.0186456 (PMC5728559; doi:10.1371/journal.pone.0186456)
Supplement: S1 File — (DOCX) [file pone.0186456.s009.docx]

**Supplemental Material: Genome-Wide Association Meta-Analysis of Fish and EPA+DHA Consumption in 17 US and European Cohorts**

**S1 Table**. Description of cohorts from the CHARGE consortium.

| **Cohort** | **Study description ^1^** | **Web Link and Relevant Reference** |
| --- | --- | --- |
| Atherosclerosis Risk in Communities (ARIC)  USA | The ARIC study is a population-based cohort study designed to study of new and established risk factors for atherosclerosis and community trends in coronary heart disease. In 1987-89, baseline data was collected on 15,792 adults, aged 45–64 y, living in four U.S. communities (Forsyth County, NC; Jackson, MI; northwest Minneapolis suburbs, MN; Washington County, MD). The baseline exam was conducted in 1987-89 and information was collected on African Americans, Whites, and a few adults of other ethnicities, aged 45–64 y. After providing informed consent, 15,792 adults were enrolled (8,710 women and 7,082 men). A total of 7,201, Caucasian adults with available DNA, valid dietary information, and consent to share genetic data were eligible for the current analysis. | [http://www.cscc.unc.edu/aric/  Am J Epidemiol. 129(4): 687-702, 1989 (4)](http://www.cscc.unc.edu/aric/Am%20J%20Epidemiol.%20129(4):%20687-702,%201989%20(4)) |
| Cardiovascular Health Study (CHS)  USA | The CHS is a prospective population-based cohort study of people ≥ 65 years old at baseline initiated to evaluate risk factors for the development and progression of cardiovascular disease. Participants were recruited at four field centers (Forsyth County, NC; Sacramento County, CA; Washington County, MD; Pittsburgh, PA)]. The baseline exam was conducted in 1989-1990. Overall, 5,201 individuals were recruited from random samples of Medicare eligibility lists. A total of 2,765 adults with available DNA, valid dietary information, and consent to share genetic data were eligible for the current analysis. | http://www.chs-nhlbi.org/ Ann Epidemiol. 1(3): 263-276, 1991 (1) |
| Dietary, Lifestyle, and Genetic determinants of Obesity and Metabolic syndrome (DILGOM). Finland | The National FINRISK 2007 Study was carried out in year 2007 in five geographical areas in Finland: the cities of Helsinki and Vantaa (the metropolitan area), the areas of Turku and Loimaa, and the provinces of North Savo, North Karelia, and Oulu. The sample of 10000 men and women aged 25-74 years was a random sample from the Finnish Population Information System, stratified according to sex, 10-year age groups, and the five geographical areas. The survey protocol followed standardized international protocols (Tolonen et al.). Survey included a self-administered questionnaire on socio-demographic factors, health behavior, and medical history, and a health examination, where anthropometric measurements, blood pressure measurements and blood sampling were carried out. The participants who took part in the first phase of the National FINRISK 2007 Study were invited to a more detailed examination on the Dietary, Lifestyle and Genetic Determinants of Obesity and Metabolic syndrome Study (the DILGOM Study) conducted between April and June in 2007. The DILGOM participants have been carefully assessed for their diet (a self-administered food frequency questionnaire (FFQ)), physical activity, psychosocial factors, markers of obesity and glucose metabolism. For the present study, valid dietary and genetic data was available for 604 (GWA genotype data) and 3467 (CardioMetabochip genotype data) individuals. | Konttinen H, Männistö S, Sarlio-Lähteenkorva S, Silventoinen K, Haukkala A. Appetite. 2010 Jun;54(3):473-9.   Inouye M, Silander K, Hämäläinen E, Salomaa V, Harald K, Jousilahti P, Männistö S, Eriksson JG, Saarela J, Ripatti S, Perola M, van Ommen GJ, Taskinen MR, Palotie A, Dermitzakis ET, Peltonen L. PLoS Genet. 2010 Sep 9;6(9).   Peltonen, M., Harald, K., Männistö, S., Saarikoski, L., Peltomäki, P., Lund, L., et al. (2008). The National FINRISK 2007 Study. Helsinki: Publications of the National Public Health Institute, B 34/2008. Available in http://www.ktl.fi/attachments/finriski/2008b34.pdf |
| Estonian Biobank | The Estonian Biobank cohort is a volunteer-based sample of the Estonian resident adult population (aged ≥18 years). At baseline, the general practitioners and medical personnel performed a standardised health examination of the participants, who also donated blood samples for DNA, white blood cells and plasma tests and filled out a 16-module questionnaire on health-related topics such as lifestyle, diet and clinical diagnoses described in WHO ICD-10. | Nelis M. et al. Genetic Structure of Europeans: A View from the North–East. PLoS ONE (2009) 4(5): e5472.  Leitsalu L, Haller T, Esko T, Tammesoo ML, Alavere H, Snieder H, Perola M, Ng PC, Mägi R, Milani L, Fischer K, Metspalu A. Cohort Profile: Estonian Biobank of the Estonian Genome Center, University of Tartu. Int J Epidemiol. (2014) Feb 11. |
| Family Heart Study (FamHS)  USA | The FHS began in 1992 with the ascertainment of 1,200 families (50% randomly sampled, and 50% high risk for CHD). The families (~6,000 individuals,) were sampled on the basis of information on probands from four population-based parent studies: the Framingham Heart Study, the Utah Family Tree Study, and two ARIC centers (Minneapolis, and Forsyth County, NC). Approximately eight years later, study participants belonging to the largest pedigrees were invited for a second clinical exam. A total of 2,767 participants of European descent in 510 extended families were examined. A total of 2,094 adults with available DNA and who provided valid dietary information were eligible for the current study. | [https://dsgweb.wustl.edu/PROJECTS/MP1.html  Higgins et al. Am J Epidemiol. 143 (12): 1219, 1996 (5)](https://dsgweb.wustl.edu/PROJECTS/MP1.htmlHiggins%20et%20al.%20%20Am%20J%20Epidemiol.%20143%20(12):%201219,%201996%20(5)) |
| Framingham Heart Study (FHS)  USA | The Framingham Offspring Study is a community-based longitudinal study designed to examine CVD risk in the offspring of the original participants and their spouses of the Framingham Heart Study cohort. In 1971, 5,124 individuals were enrolled in the study; since then, the cohort has been examined every 3–4 y. Between 1991 and 1995, during the 5th examination cycle, 3,799 adults, with a mean age of 54.98, underwent a standardized medical history and physical examination. Beginning in 2002, 4,095 Gen III participants, who had at least one parent in the offspring cohort, were enrolled in the Framingham Heart Study. At the first cycle of the Gen III study, 4,095 individuals with a mean age of 40 y, underwent the standard clinic examination. For the present study both cohorts were combined for the analysis. A total of 5,835 adults with available DNA, valid dietary information, and consent to share genetic data were eligible for the current study. | [http://www.framinghamheartstudy.org/  Prev Med.4:518–25, 1975 (2) Am J Epidemiol. 165(11):1328-35, 2007 (3)](http://www.framinghamheartstudy.org/Prev%20Med.4:518–25,%201975%20(2)Am%20J%20Epidemiol.%20165(11):1328-35,%202007%20(3)) |
| Health, Aging and Body Composition  (Health ABC).  USA | The Health ABC study is a prospective cohort study investigating the associations between body composition, weight-related health conditions, and incident functional limitation in older adults. Health ABC enrolled well-functioning, community-dwelling black (n=1281) and white (n=1794) men and women aged 70-79 years between April 1997 and June 1998. Participants were recruited from a random sample of white and all black Medicare eligible residents in the Pittsburgh, PA, and Memphis, TN, metropolitan areas. Participants have undergone annual exams and semi-annual phone interviews. A total of 1,249 Caucasian participants who attended the second exam in 1998-1999, and who had available genotyping and food frequency data were eligible for the current study. | <http://www.nia.nih.gov/ResearchInformation/ScientificResources/HealthABCDescription.htm> |
| Health Professionals Follow-up Study | The HPFS was initiated in 1986 when 51,529 male health professionals between 40 and 75 years of age years and residing in the U.S. completed an FFQ and a questionnaire on lifestyle and medical history. The participants have been followed with repeated questionnaires on lifestyle and health every 2 years and FFQs every 4 years. Participants for the current study were those with information on fish consumption and genome-wide scan data. |  |
| Helsinki Birth Cohort Study (HBCS) | HBCS: Helsinki Birth Cohort Study (HBCS) includes 8,760 subjects born in Helsinki between 1934 and 1944. Between 2000 and 2003, a representative subset of 928 males and 1,075 females participated in a clinical study focusing upon cardiovascular and metabolic outcomes, cognitive function and psychological outcomes. A total of 1302 subjects with available DNA, valid dietary information, and consent to share genetic data were eligible for the current study. | Barker DJ, Osmond C, Forsén TJ, Kajantie E, Eriksson JG. Trajectories of growth among children who have coronary events as adults. N Engl J Med. 2005 Oct 27;353(17):1802-9 |
| Health 2000 Study | The Health 2000 survey (H2000), originally designed to provide information on health of the Finnish population, was a nationally representative sample of Finns aged 30 or over and it was carried out from fall 2000 to spring 2001. We obtained a subset of the survey participants for our genetic study by identifying all those who fulfilled the IDF definition of metabolic syndrome criteria, and selecting a matched control for each (previously described by Pajunen et al). A total of 1935 individuals with available genotype data and valid dietary information were eligible for the current study. | http://www.terveys2000.fi/indexe.html   HEALTH AND FUNCTIONAL CAPACITY IN FINLAND, Baseline Results of the Health 2000 Health Examination Survey. In: Aromaa A, Koskinen S, eds. Vol KTL B12/2004. Helsinki: National Public Health Institute; 2004.    Pajunen P, Rissanen H, Härkänen T, Jula A, Reunanen A, Salomaa V. Diabetes Metab. 2010;36:395-401. |
| Invecchiare in Chianti (aging in the Chianti area, InCHIANTI)  Italy | InCHIANTI is a population-based study designed to evaluate the factors that influence mobility in older people in the Chianti region of Tuscany, Italy. A total of 1,616 residents were selected from the population registry of Greve (a rural area: 11,709 residents with 19.3% of the population greater than 65 years of age), and Bagno a Ripoli (Antella village near Florence; 4,704 inhabitants, with 20.3% greater than 65 years of age). The participation rate was 90% (n=1453), and the participants ranged between 21-102 years of age. For the present study, 1,071 adults with available DNA and who provided complete dietary information were eligible for the current study. | [http://www.inchiantistudy.net/bindex.html  Ferrucci L, et al. J Am Geriatr Soc. 48:1618-1625, 2000 (12)](http://www.inchiantistudy.net/bindex.htmlFerrucci%20L,%20et%20al.%20J%20Am%20Geriatr%20Soc.%2048:1618-1625,%202000%20(12)) |
| Multi-Ethnic Study of Atherosclerosis (MESA) | The MESA is a cohort study designed to investigate the characteristics of subclinical cardiovascular disease and the risk factors that predict progression to clinically overt cardiovascular disease or progression of the subclinical disease. MESA comprises a diverse, population-based sample of 6,814 asymptomatic men and women aged 45-84. Thirty-eight percent of the recruited participants are Caucasian, 28 percent African-American, 22 percent Hispanic, and 12 percent Asian, predominantly of Chinese descent. Participants were recruited from six field centers across the United States: Wake Forest University, Columbia University, Johns Hopkins University, University of Minnesota, Northwestern University and University of California - Los Angeles. In the current analysis only data from Caucasian participants were analyzed, including up to 2302 unrelated adults with valid dietary information and consent to share genetic data. | [http://www.mesa-nhlbi.org/  Bild DE, Bluemke DA, Burke GL, Detrano R, Diez Roux AV, Folsom AR, Greenland P, Jacob DR,Jr, Kronmal R, et al. Multi-ethnic study of atherosclerosis: Objectives and design. Am J Epidemiol. 2002 Nov 1;156(9):871-81.](http://www.mesa-nhlbi.org/Bild%20DE,%20Bluemke%20DA,%20Burke%20GL,%20Detrano%20R,%20Diez%20Roux%20AV,%20Folsom%20AR,%20Greenland%20P,%20Jacob%20DR,Jr,%20Kronmal%20R,%20et%20al.%20Multi-ethnic%20study%20of%20atherosclerosis:%20Objectives%20and%20design.%20Am%20J%20Epidemiol.%202002%20Nov%201;156(9):871-81.) |
| Nurse's Health Study | The NHS was established in 1976 when 121,700 female registered nurses aged 30-55 years and residing in 11 large U.S. states completed a mailed questionnaire on medical history and lifestyle characteristics (PMID 15864280). Every two years, follow-up questionnaires have been sent to update information on exposures and newly diagnosed diseases and every 2 to 4 years diet was assessed using a validated semi-quantitative FFQ (Willett, Nutritional Epidemiology). Participants for the current study were those with information on fish consumption and genome-wide scan data. |  |
| Rotterdam Study Netherlands | The Rotterdam Study a prospective cohort study among, initially, 7,983 persons living in Rotterdam in The Netherlands (78% of 10,215 invitees). The first cohort (RS-I) started in 1990 in Ommoord, a suburb of Rotterdam, the Netherlands, comprising of 7,983 men and women aged 55 years and over. Baseline measurements were obtained between 1990 and 1993. Trained research assistants collected data on current health status, use of medication, medical history, lifestyle and risk indicators for chronic diseases during an extensive home interview. Subsequently, the participants visited the study center for detailed clinical examinations. Follow up visits were held every 2-3 years. | Hofman, Albert, Sarwa Darwish Murad, Cornelia M. van Duijn, Oscar H. Franco, André Goedegebure, M. Arfan Ikram, Caroline C. W. Klaver, et al. “The Rotterdam Study: 2014 Objectives and Design Update.” Eur J Epidemiol (2013) 28:889–926  http://www.erasmus-epidemiology.nl/research/ergo.htm |
| The Hellenic Study of Interactions between SNPs and Eating in Atherosclerosis Susceptibility (THESIAS) | The Hellenic Study of Interactions between Snps and Eating in Atherosclerosis Susceptibility (THISEAS) study is a case- control study designed to investigate the association between genetic and lifestyle environmental factors and the risk of coronary artery disease in men and women aged >25 yrs. The control group consists of individuals with no history of cardiovascular disease, while cases are individuals with coronary artery disease. Hematological, biochemical and anthropometric measurements were conducted to all participants. Dietary assessment and physical activity data were collected through face to face interview by well trained scientists. Exclusion criteria for the control group were history of cardiovascular disease, cancer and/ or other inflammatory disease. The population for the present analysis was comprised of 395 subjects with phenotype, genotype and dietary data available. | PMID:20167083 |
| Women's Genome Health Study | The Women’s Genome Health Study (WGHS) is a prospective cohort of initially healthy, female North American health care professionals at least 45 years old at baseline representing participants in the Women’s Health Study (WHS) who provided a blood sample at baseline and consent for blood-based analyses. The WHS was a 2x2 trial beginning in 1992-1994 of vitamin E and low dose aspirin in prevention of cancer and cardiovascular disease with about 10 years of follow-up. Since the end of the trial, follow-up has continued in observational mode. Additional information related to health and lifestyle were collected by questionnaire throughout the WHS trial and continuing observational follow-up. | Ridker PM, Chasman DI, Zee RY, Parker A, Rose L, Cook NR, Buring JE; Women's Genome Health Study Working Group. Rationale, design, and methodology of the Women's Genome Health Study: a genome-wide association study of more than 25,000 initially healthy american women. Clin Chem. 2008 Feb;54(2):249-55.PMID: 18070814 |
| YFS | The Cardiovascular Risk in Young Finns (YFS) is a population-based 27 year follow up-study. The first cross-sectional survey was conducted in 1980, when 3,596 Caucasian subjects aged 3-18 years participated. In adulthood, the 27-year follow-up study was conducted in 2007 (ages 30-45 years) with 2,204 participants. The study cohort for the present analysis comprised subjects who had participated in the study in 2007 and had validated dietary data from FFQ, available genotype and other risk factor data. The study was approved by the local Ethical Committees and was performed according to Helsinki declaration. | [http://youngfinnsstudy.utu.fi/ Raitakari OT et al. Cohort profile. Int. J Epidemiol. 2008;37:1220-6](http://youngfinnsstudy.utu.fi/Raitakari%20OT%20et%20al.%20Cohort%20profile.%20Int.%20J%20Epidemiol.%202008;37:1220-6) |

**Supplemental Material: Genome-Wide Association Meta-Analysis of Fish and EPA+DHA Consumption in 17 US and European Cohorts**

**S2 Table**. Dietary assessment methods for CHARGE cohorts.

| **Study** | **Description** | **FFQ Line items / Top contributing Food Groups** |
| --- | --- | --- |
| Atherosclerosis Risk in Communities (ARIC) USA | An interviewer-administered, 66-item semi-quantitative FFQ that was modified from the validated Willett 61-item FFQ (19) (modifications described elsewhere (20)). Participants were asked to indicate how often, on average, they consumed various foods and beverages over the past year according to 9 frequency categories, ranging from never or <1 time/mo to ≥6 times/d. Standard portion sizes given as a reference for intake estimation. Supplementary questions included regarding frequency of fried food consumption and brand name of the breakfast cereal most commonly consumed (open-ended response). Dietary information was judged as unreliable and excluded from further analysis if total energy intake was estimated to be <500 or >3600 kcal for women and <600 or >4200 kcal for men or if 10 or more items of the FFQ were unanswered.  Related References: Willett WC, et al. Am J Epidemiol. Jul 1985;122(1):51-65. (19) Stevens J et al. Nutrition Research 1996;16: 735-745. (20) | 4 items:  canned tuna fish; dark meat fish; other fish; shrimp, lobster, scallops |
| Cardiovascular Health Study (CHS) USA | Usual dietary intake was assessed using a picture-sort version of the National Cancer Institute FFQ. This is a 99-item, self-administered FFQ. Participants were asked to indicate how often, on average, they consumed various foods and beverages over the past year according to 9 frequency categories, ranging from never to >5 times per week.. Portion sizes were illustrated by color pictures or laminated 4 X 6 in (10 X 15 cm) index card with a black-and white line drawing. Dietary information was judged as unreliable and excluded from further analysis if calculated total kilocalories were < 500 or > 5000 kcal/d. Related References: Kumanyika S, et al. J Am Diet Assoc. 1996 Feb;96(2):137-44. (16) | 2 line items: 1) tuna fish/tuna salad/tuna casserole and 2) other fish, broiled or baked. |
| Dietary, Lifestyle, and Genetic Determinants of Obesity and Metabolic Syndrome (DILGOM) | Food consumption over the previous 12 months was assessed with a validated self-administered FFQ updated for this study. The average use of 132 food items and mixed dishes were recorded by nine frequency categories ranged from never or seldom to at least six times a day. The portion size was fixed for each food item and mixed dish (e.g., slice and glass). Reporting additional items consumed frequently but not listed in the FFQ was also allowed. The participants completed the FFQ at the study site, where a trained study nurse reviewed the questionnaire. Exclusions were made due to incompletely filled FFQs (n = 74) and daily energy intake cut-offs corresponding to 0.5 % at both ends of the daily energy intake distributions for men and women (n = 48). The average daily intakes of food groups and nutrients were calculated by the national food composition database, Fineli (http://www.fineli.fi/index.php), using in-house software. References: Männistö S, Virtanen M, Mikkonen T, Pietinen P. Reproducibility and validity of a food frequency questionnaire in a case-control study on breast cancer. J Clin Epidemiol 1996;49:401-409. Paalanen L, Männistö S, Virtanen MJ, Knekt P, Räsänen L, Montonen J, Pietinen P. Validity of a food frequency questionnaire varied by age and body mass index. J Clin Epidemiol. 2006 Sep;59(9):994-1001 Reinivuo H, Hirvonen T, Ovaskainen ML, Korhonen T, Valsta LM. Dietary survey methodology of FINDIET 2007 with a risk assessment perspective. Public Health Nutr 2010;13:915-919. | Fish soup Frozen fish or fish fingers Salmon or rainbow trout Baltic herring Pikeperch, whitefish, perch, vendace or pike Smoked fish (e.g. whitefish, salmon or rainbow trout) Spiced or salted fish Tuna or other canned fish Kalakukko [rye bread fish pasty] Shrimp or crayfish |
| Estonian Biobank | A questionnaire administered by trained interviewers. Participants were asked to indicate how often, on average, they consumed various foods and beverages over the past year according to 4 frequency categories: never; 1-2 times/week; 3-5 times/week, ≥6 times/week. |  |
| Family Heart Study (FamHS) USA | A 66-item questionnaire modified from the Willet FFQ administered by trained interviewers. Participants were asked to indicate how often, on average, they consumed various foods and beverages over the past year according to 9 frequency categories, ranging from never or <1 time/mo to ≥6 times/d. Portion sizes were specified. Dietary information was judged as unreliable and excluded from further analysis if reported energy intakes were <3347.2kJ/day (799.3 kcal/day) or >17572.8 kJ/day (4196.4 kcal/day) for men and <2510.4 kJ/d (599.5 kcal/day) or >14644 kJ/day (3497 kcal/day) for women.  Related References: Stein AD et al. Am J Epidemiol 1992;135(6):667-677. (21) Willett WC, et al. Am J Epidemiol. Jul 1985;122(1):51-65. (19) | 4 items:  canned tuna fish dark meat fish (salmon, mackerel, swordfish, sardines, bluefish) Other fish (cod, perch, catfish, etc) Shrimp, lobster, scallops |
| Framingham Heart Study (FHS) USA | A self-administered 126-item FFQ. Participants were asked to indicate how often, on average, they consumed various foods and beverages over the past year according to 9 frequency categories, ranging from never or <1 time/mo to ≥6 times/d. Portion sizes were specified. Separate questions about the use of vitamin and mineral supplements and the type of breakfast cereal most commonly consumed were also included in the FFQ. Dietary information was judged as unreliable and excluded from further analysis if reported energy intakes were < 2.51 MJ/d (600 kcal/d) or > 16.74 MJ/d (4000 kcal/d) for women and > 17.57 MJ/d (4200 kcal/d) for men or if >= 12 food items were left blank.  Related References: Rimm et al. Am J Epidemiol 1992;135:1114–26, 1127–36. (17) Salvini S et al. Int J Epidemiol 1989;18:858–67. (18) | 4 items:  1. canned tuna fish 2. dark meat fish e.g. mackerel, salmon, sardines, bluefish, swordfish) 3. other fish 4. shrimp, lobster, scallops as a main dish |
| Helsinki Birth Cohort Study (HBCS) | Diet was assessed with a validated, self-administered 128-item FFQ. The FFQ was designed to assess the ordinary diet over the previous 12 mo. The subjects were asked to indicate the average intake frequency of each food item and mixed dish. The 9 possible frequency categories ranged from never or seldom to ≥6 times/d. The portion sizes were fixed, eg, a glass or a slice of bread. Food and nutrient intake was calculated using the Finnish Food Composition Database, Fineli. Dietary information was judged as unreliable and excluded from further analysis if reported energy intakes were <650 or >6100 kcal/d, corresponding to 0.5% at each end of the selfreported daily energy intake scale or if ≥12 food items were left blank. Related References: Männistö et al. J Clin Epidemiol 1996;49:401-9.  Paalanen et al. J Clin Epidemiol 2006;59:994-1001. | Fish soup Frozen fish or fish fingers Salmon or rainbow trout Baltic herring Pikeperch, whitefish, perch, vendace or pike Spiced or salted fish Tuna or other canned fish Kalakukko (rye bread fish pasty) Shrimp or crayfish |
| Health, Aging and Body Composition (Health ABC). USA | A 108-item interviewer-administered FFQ (Block Dietary Data Systems, Berkeley, CA). Participants were asked to indicate how often, on average, they consumed various foods and beverages over the past year according to nine frequency categories, ranging from “never” to “every day”. Portion size information was collected by trained interviewers using wood blocks, food models, standard kitchen measures, and flash cards to help participants estimate portion sizes. Individuals with serious errors (skipped >15% of items or reported <3 or >20 foods/day) on the FFQ and those who reported energy intakes less than 500 kcal/d or greater than 3,500 kcal/d in women and less than 800 kcal/d or greater than 4,000 kcal/d in men were excluded.  Related References: Houston et al. Am J Clin Nutr. 2008; 87(1):150-5. (15) | 4 items -- 1: shellfish like shrimp, scallops, crab 2: tuna, tuna salad, tuna casserole 3: fried fish or fish sandwich 4: other fish, broiled or baked |
| HPFS | Fish intake was assessed as described for NHS. For the present analysis, we included the participants mean total fish intakes of the 1986 and 1990 FFQs. | 1: canned tuna fish 2: dark meat fish 3: other fish 4: shrimp, lobster, scallops as a main dish |
| H2000 | Dietary data were collected using food frequency questionnaire of the preceding year. It consisted of 128 commonly used or nutrionally important food items and mixed dishes. Validity of this FFQ meets the requirements of epidemiological studies (1, 2). The items were grouped under 12 sub-headings one of them being fish dishes. Nine frequency categories ranged from “never or rarely” to “six or more times per day”. The portion sizes were fixed and if possible, specified using natural units (e.g. serving slice, glass, cup). Fish consumption was converted into grams per day by multiplying the food consumption frequency by fixed portion sizes. Food and nutrient intake was calculated using the Finnish Food Composition Database (http://www.fineli.fi/index.php). Fasting blood samples were collected to measure serum concentrations of fatty acids in a Health 2000 Sub-study (3). Serum fatty acids composition was analyzed using a gas chromatograph (4). The fish-derived long-chain n-3 fatty acids (eicosapentaenoic acid, docosahexaenoic acid and docosapentaenoic acid) were expressed in mg/L and as proportions from total fatty acids. References: 1. Paalanen L, Männistö S, Virtanen MJ, Knekt P, Räsänen L, et al. (2006) Validity of a food frequency questionnaire varied by age and body mass index. J Clin Epidemiol 59: 994–1001. 2. Männistö S, Virtanen M, Mikkonen T, Pietinen P (1996) Reproducibility and validity of a food frequency questionnaire in a case-control study on breast cancer. J Clin Epidemiol 49: 401–409. 3. Suominen-Taipale AL, Partonen T, Turunen AW, Männistö S, Jula A, Verkasalo PK. Fish consumption and omega-3 polyunsaturated fatty acids in relation to depressive episodes: a cross-sectional analysis.PLoS One. 2010 May 7;5(5):e10530. 4. Jula A, Marniemi J, Rönnemaa T, Virtanen A, Huupponen R (2005) Effects of diet and simvastatin on fatty acid composition in hypercholesterolemic men: a randomized controlled trial. Arterioscler Thromb Vasc Biol 25: 1952–1959. | Fish soup Frozen fish or fish fingers Salmon or rainbow trout Baltic herring Pikeperch, whitefish, perch, vendace or pike Spiced or salted fish Tuna or other canned fish Kalakukko (rye bread fish pasty) Shrimp or crayfish |
| Invecchiare in Chianti (aging in the Chianti area, InCHIANTI) Italy | A 236 item, interviewer administered FFQ that investigates how frequently (weekly, monthly, yearly) each specific food was generally consumed. Participant is asked to specify the size of the portion usually consumed, in comparison to a range of portion that are shown in colored photographs. Nutrient data for specific foods were obtained from the Food Composition Database for Epidemiological Studies in Italy (18). Dietary information was judged as unreliable and excluded from further analysis if reported energy intakes less than 600 kcal/d or greater than 4,000 kcal/d and 4,200 kcal/d in women and men, respectively.  Related References: Bartali et al. Arch. Gerontol Geriatr. Geriatr. 38 2004; 51–60. (30) Pisani et al. Int J Epidemiol. 1997; 26:152–160. (31) | salted fish, canned fish, cod, flounder, sardines, trout, sword fish, other kind of fish |
| Multi-Ethnic Study of Atherosclerosis (MESA) | 120-item, self-administered, modified-Block FFQ [Mayer-Davis E et al. Ann Epidemiol 1999;9:314–324. & Nettleton JA et al. Br J Nutr 2009; 102, 1220–1227.] | shrimp, lobster, crab, oysters, mussels (not fried); tuna, salmon, sardines (including sashimi or sushi); other broiled, steamed, baked or raw fish (trout, sole, halibut, poke, grouper); fried fish or fish sandwich, fried shrimp, calamari; fish stew or seafood gumbo, paella; stir-fried shrimp or fish with vegetables; |
| Nurse's Health Study | For the present analysis, we included the participants’ mean total fish intakes of the 1984 and 1986 FFQs. The FFQ included four line items for canned tuna, dark fish, other fish and seafood main dishes. For each item, participants were asked how often, on average, they had consumed a specified amount of each food over the past year. The participants could choose from nine frequency categories (never, 1-3 per month, 1 per week, 2-4 per week, 5-6 per week, 1 per day, 2-3 per day, 4-5 per day and 6 or more per day). We assessed the total number of fish servings/d by summing across line items. | 1: canned tuna fish 2: dark meat fish 3: other fish 4: shrimp, lobster, scallops as a main dish |
| Rotterdam Study Netherlands | Dietary assessment followed a two-step procedure: 1) A simple self-administered questionnaire was first completed at home, only questions were asked about which food items were consumed; no questions about portion sizes (or frequency) were asked during this step. 2) A subsequent structured interview was later conducted at the research center with a trained dietitian Participants were asked to indicate how often, on average, they consumed various foods and beverages over the past year according to 9 frequency categories, ranging from never or <1 time/mo to ≥6 times/d. Portion sizes were presented in natural units (eg. slices of bread) or household measures (e.g., cups, bowls, tablespoons, plates, etc.) Nutritional supplement intakes were not considered because dose and duration were not recorded with sufficient accuracy. Dietary information was judged as unreliable and excluded from further analysis if a dietician considered the reported dietary intake unreliable, i.e. because participant's answers during the dietary interview were either too inconsistent or too incomplete. Related References: Klipstein-Grobusch K, et al. Eur J Clin Nutr. 1998 Aug; 52(8):588-96. (29) | Eel ; Fish lean 0-2 g fat ; Fish medium fat ; Fish fat > 10 g fat ; Herring salted ; Mackerel ; Sardines ; Pilchards in oil canned ; Salmon canned ; Eel smoked ; Plaice ; Fish fingers ; Cod ; Herring marinated |
| The Hellenic Study of Interactions between SNPs and Eating in Atherosclerosis Susceptibility (THESIAS) | Dietary assessment data was collected through face to face interview by well trained scientists. A semi- quantitative 172- item questionnaire was used to assess dietary intake. Participants were asked to indicate how often they consumed various foods and beverages, as well as the portion size by comparison with photos. Daily consumption was calculated from the FFQ by multiplying the standard serving size of each food (as described by the Ministry of Health and Welfare, Supreme Scientific Health Council) by the value corresponding to each consumption frequency: never; 1–3 times/month ; 1–2 times/week; 3–4 times/week; 5-6 times/week; 1time/day. Related References: Ministry of Health and Welfare, Archives of Hellenic Medicine 1999, 16(5): 516-524 | Canned fish (tuna, salmon etc), Small Fatty Fish fresh or frozen (sardine, anchovy etc), Other small fish fresh or frozen (smelts), Big fatty fish fresh or frozen(salmon, mackerel etc), Other big fish fresh or frozen, Seafood |
| Women's Genome Health Study (WGHS) | Dietary omega-3 fatty acids were derived from a previously validated baseline 128-food-frequency questionnaire. Fish consumption was assessed through 4 items on the FFQ. Participants were asked to report their average consumption of canned tuna (3–4 oz), dark-meat fish (3–5 oz), other fish (3–5 oz), and shrimp, lobster, and scallops as a main dish over the past year. Possible responses included never or <1 time/mo, 1–3 times/mo, 1 time/wk, 2–4 times/wk, 5–6 times/wk, 1 time/d; 2–3 times/d, 4–5 times/d, and ≥6 times/d. Individual responses were converted into servings per day by using the midpoint for each response category. The fish variable (servings/day) was created by the summing the frequency of consumption of canned tuna, dark fish, other fish, and shrimp, lobster, and scallops as a main dish. Related references:  Willett WC, Sampson L, Stampfer MJ, Rosner B, Bain C, Witschi J, Hennekens CH, Speizer FE. Reproducibility and validity of a semiquantitative food frequency questionnaire. Am J Epidemiol. 1985 Jul;122(1):51-65. PubMed PMID: 4014201 Djoussé L, Gaziano JM, Buring JE, Lee IM. Dietary omega-3 fatty acids and fish consumption and risk of type 2 diabetes. Am J Clin Nutr. 2011 Jan;93(1):143-50. PMID: 20980491 | 1: canned tuna fish 2: dark meat fish 3: other fish 4: shrimp, lobster, scallops as a main dish |
| Young Finns Study (YFS) | Dietary data were collected using a 131-item food frequency questionnaire, self-administered and checked by a nurse. Participants were asked to report their food consumption during the previous 12 months. The questionnaire had fixed portion sizes and 9 response categories from “never or rarely” to “6 or more times per day”. Related references: 1. Paalanen L, Männistö S, Virtanen MJ, Knekt P, Räsänen L, et al. Validity of a food frequency questionnaire varied by age and body mass index. J Clin Epidemiol 2006;59: 994–1001. 2. Männistö S, Virtanen M, Mikkonen T, Pietinen P. Reproducibility and validity of a food frequency questionnaire in a case-control study on breast cancer. J Clin Epidemiol 1996;49:401–409. | Fish soup Frozen fish or fish fingers Salmon or rainbow trout Baltic herring Pikeperch, whitefish, perch, vendace or pike Spiced or salted fish Tuna or other canned fish Kalakukko (rye bread fish pasty) Shrimp or crayfish |

**Supplemental Material: Genome-Wide Association Meta-Analysis of Fish and EPA+DHA Consumption in 17 US and European Cohorts**

**S3 Table3.** Genotyping Methods for CHARGE cohorts

|  |  | **ARIC** | **CHS** | **DILGOM** | **Estonian Study** | **FamHS** |
| --- | --- | --- | --- | --- | --- | --- |
|  | Array | Affymetrix 6.0 | Illumina HumanCNV370-Duo BeadChip | Illumina BeadChip Human 610-Quad | Illumina 370CNV, Illumina OmniExpress | Illumina 510, 650, and 1M |
|  | Genotype Calling software | Birdseed | Illumina BeadStudio | Illuminus | Illumina BeadStudio | Illumina BeadStudio Suite |
|  | Sample Size | 9557 | 3230 | 604 | 9920 | 3523 |
| Genotyping QC | Exlusions (subject): |  |  |  |  |  |
|  | Call rate | < 95% | < 95% | < 95% | < 95% | < 98% |
|  | other | sex mismatch, first-degree relative, genetic outlier | duplicate, Mendelian inconsistensies, heterozygote frequency=0 | heterozygosity, gender check and relatedness checks | heterozygosity, gender check and relatedness checks | Mendelian inconsistencies |
|  | Exlusions (SNP): |  |  |  |  |  |
|  | Call rate | < 95% | < 95% | < 95% | < 98% | < 98% |
|  | MAF | < 1% | n.a. | < 1% | < 1% | < 1% |
|  | HWE | < 10^-5^ | < 10^-5^ | NA | < 10^-6^ | < 10^-6^ |
| Imputation information | Imputation program | MACH | BIMBAM10 v0.91 | MACH 1.0 (Hapmap r22 CEU) | IMPUTE | MACH (v 1.0.16) |
|  | NCBI build for Imputation | 36 | 35 | 36 | 36 | 36 |
|  | analysis program | ProbABLE | R | ProbABEL | ProbABEL | SAS, R |

**S3 Table, cont’d.** Genotyping Methods for CHARGE cohorts

|  |  | **FHS** | **Health ABC** | **HPFS_T2D** | **HPFS_CHD** | **HPFS_KS** |
| --- | --- | --- | --- | --- | --- | --- |
|  | Array | Affymetrix 500K and MIPS 50K | Illumina Human 1M-Duo BeadChip | Affymetrix 6.0 | Affymetrix 6.0 | Illumina 610Q |
|  | Genotype Calling software | BRLMM | Birdseed | BirdSeed | BirdSeed | Beadstudio |
|  | Sample Size | 6371 | 1497 | 2456 | 1133 | 547 |
| Genotyping QC | Exlusions (subject): |  |  |  |  |  |
|  | Call rate | < 97% | <97% | <=98% | <=98% | < 95% |
|  | other | Heterozygosity filter 5SD from eman, > 1000 Mendelian errors | sample failure, sex mismatch, first-degree relative | -sex discrepancy with genetic data from X-linked markers -duplicates and first/second degree relatives -PCA outliers -heterozygosity -autosomal chromosome abberations -missing phenotype & covariate information | -duplicates and first/second degree relatives -PCA outliers -heterozygosity -missing phenotype & covariate information | -duplicates and first/second degree relatives -PCA outliers -missing phenotype & covariate information |
|  | Exlusions (SNP): |  |  |  |  |  |
|  | Call rate | < 95% | < 97% | <=98% | <=98% | < 95% |
|  | MAF | < 1% | < 1% | < 2% | < 2% | < 1% |
|  | HWE | < 10^-6^ | < 10^-6^ | <1e-4 | <1e-4 | <1e-5 |
| Imputation information | Imputation program | MACH | MACH 1.0.16 | MACH | MACH | MACH |
|  | NCBI build for Imputation | release 22,build 36 | 36 | 36 | 36 | 36 |
|  | analysis program | R | R | ProbABLE | ProbABLE | ProbABLE |

**S3 Table, cont’d.** Genotyping Methods for CHARGE cohorts

|  |  | **HBCS** | **H2000** | **InCHIANTI** | **MESA** | **NHS_T2D** |
| --- | --- | --- | --- | --- | --- | --- |
|  | Array | modified Illumina 610k | Illumina Human610-Quad BeadChip | Illumin 550K | Affymetrix 6.0 | Affymetrix 6.0 |
|  | Genotype Calling software | Illumina BeadStudio | Illuminus | Beadstudio | Birdseed | BirdSeed |
|  | Sample Size | 1701 | 1935 | 1194 | 2302 | 3145 |
| Genotyping QC | Exlusions (subject): |  |  |  |  |  |
|  | Call rate | < 95% | < 95% | < 98.5 | < 95% | <=98% |
|  | other | sex mismatch, first-degree relative, genetic outlier | heterozygosity, gender check and relatedness checks | sex mispecifications, heterozygosity > 0.3, duplicate | Heterozygosity < 53% | -sex discrepancy with genetic data from X-linked markers -duplicates and first/second degree relatives -PCA outliers -heterozygosity -autosomal chromosome abberations -missing phenotype & covariate information |
|  | Exlusions (SNP): |  |  |  |  |  |
|  | Call rate | < 95% | < 95% | < 99 | < 95% | <=98% |
|  | MAF | < 1% | < 5% | <1% | NA | < 2% |
|  | HWE | < 10^-6^ | NA | <10^-4^ | NA | <1e-4 |
| Imputation information | Imputation program | MACH | MACH 1.0 (Hapmap r22 CEU) | MACH 1.0.16 | IMPUTE (2.1.0) | MACH |
|  | NCBI build for Imputation | release 22,build 36 | 36 | 36 | 36 | 36 |
|  | analysis program | Plink/ ProbABEL | ProbABEL | MERLIN | Plink/SNPTEST | ProbABLE |

**S3 Table, cont’d.** Genotyping Methods for CHARGE cohorts

|  |  | **NHS_CHD** | **NHS_KS** | **NHS_Cancer** | **Rotterdam** | **THISEAS** |
| --- | --- | --- | --- | --- | --- | --- |
|  | Array | Affymetrix 6.0 | Illumina 610Q | Illumina 550 | Illumina 550K | OmniExpress |
|  | Genotype Calling software | BirdSeed | Beadstudio | Beadstudio | BeadStudio | Illuminus |
|  | Sample Size | 1102 | 478 | 2054 | 4606 | 1075 |
| Genotyping QC | Exlusions (subject): |  |  |  |  |  |
|  | Call rate | <=98% | < 95% | <=90% | < 97.5% | <95% |
|  | other | -duplicates and first/second degree relatives -PCA outliers -heterozygosity -missing phenotype & covariate information | -duplicates and first/second degree relatives -PCA outliers -missing phenotype & covariate information | -duplicates and first/second degree relatives -PCA outliers -missing phenotype & covariate information | excess autosomal heterozygosity, sex mismatch, genetic outlier | duplicates, heterozygosity, ethnic outliers, sex mismatch |
|  | Exlusions (SNP): |  |  |  |  |  |
|  | Call rate | <=98% | < 95% | <=90% | < 90% | <98% |
|  | MAF | < 2% | < 1% | < 1% | < 1% | na |
|  | HWE | <1e-4 | <1e-5 | n.a. | < 10^-6^ | <10-4 |
| Imputation information | Imputation program | MACH | MACH | MACH | MACH | na |
|  | NCBI build for Imputation | 36 | 36 | 36 | 36 | na |
|  | analysis program | ProbABLE | ProbABLE | ProbABLE | ProbABEL | Plink |

**S3 Table, cont’d.** Genotyping Methods for CHARGE cohorts

|  |  | **WGHS** | **Young Finns** | **DILGOM (Metabochip)** |
| --- | --- | --- | --- | --- |
|  | Array | Illumina HumanHap300 Duo+ | Illumina 670K | Illumina Metabochip |
|  | Genotype Calling software | BeadStudio v3.3 | Illuminus | GenCall |
|  | Sample Size | 23294 | 1815 | 3467 |
| Genotyping QC | Exlusions (subject): |  |  |  |
|  | Call rate | <98% | < 95% | < 95% |
|  | other | NA | duplicates, heterozygosiy, sex mismatch, relatedness | 1) heterozygosity <23.9% or >27.6%; 2) ethnic outliers;  3) related individuals and duplicates. |
|  | Exlusions (SNP): |  |  |  |
|  | Call rate | <90% | < 95% | < 95% |
|  | MAF | <1% | < 1% | < 1% |
|  | HWE | <10-6 | < 10^-6^ | < 10^-6^ |
| Imputation information | Imputation program | MACH 1.0.16 | MACH 1.0 | NA |
|  | NCBI build for Imputation | 36 | 36 | NA |
|  | analysis program | ProbABEL | ProbABEL | ProbABEL |

**Supplemental Material: Genome-Wide Association Meta-Analysis of Fish and EPA+DHA Consumption in 17 US and European Cohorts**

**S4 Table.** Associations of top Fish and EPA+DHA SNPs with circulating DHA and EPA levels*

| **SNPID** | **Effect/ Non-effect** | **DHA GWAS results** | | |  | **EPA GWAS results** | | |
| --- | --- | --- | --- | --- | --- | --- | --- | --- |
|  |  | **Effect** | **StdErr** | **P-value** |  | **Effect** | **StdErr** | **P-value** |
| *Top Fish Intake SNPs* | |  |  |  |  |  |  |  |
| rs9502823 | A/G | -0.006 | 0.063 | 0.929 |  | -0.013 | 0.018 | 0.493 |
| rs17396472 | A/T | -0.137 | 0.093 | 0.142 |  | 0.010 | 0.030 | 0.745 |
| rs1860343 | T/C | -0.009 | 0.014 | 0.520 |  | -0.007 | 0.005 | 0.126 |
| rs1562806 | T/C | -0.043 | 0.063 | 0.493 |  | -0.013 | 0.023 | 0.583 |
| rs16834168 | A/G | 0.037 | 0.057 | 0.520 |  | 0.011 | 0.016 | 0.504 |
| *Top EPA+DHA Intake SNPs* | | |  |  |  |  |  |  |
| rs11877506 | A/G | -0.045 | 0.054 | 0.406 |  | 0.009 | 0.019 | 0.652 |
| rs2456163 | T/C | -0.010 | 0.043 | 0.812 |  | -0.020 | 0.013 | 0.110 |
| rs7476409 | T/C | -0.030 | 0.049 | 0.545 |  | -0.004 | 0.015 | 0.775 |
| rs7206790 | C/G | -0.006 | 0.017 | 0.718 |  | -0.001 | 0.006 | 0.938 |

*Results from Lemaitre RN, Tanaka T, Tang W, Manichaikul A, Foy M, et al. (2011) Genetic loci associated with plasma phospholipid n-3 fatty acids: a meta-analysis of genome-wide association studies from the CHARGE Consortium. PLoS Genet 7: e1002193
